# Transient postnatal overfeeding causes liver stress-induced premature senescence in adult mice

**DOI:** 10.1038/s41598-017-11756-2

**Published:** 2017-10-10

**Authors:** Catherine Yzydorczyk, Na Li, Hassib Chehade, Dolores Mosig, Mickael Bidho, Basile Keshavjee, Jean Baptiste Armengaud, Katya Nardou, Benazir Siddeek, Mohamed Benahmed, Catherine Vergely, Umberto Simeoni

**Affiliations:** 1 Woman-Mother-Child Department, Division of Pediatrics, DOHaD Laboratory, Centre Hospitalier Universitaire Vaudois and University of Lausanne, Lausanne, Switzerland; 2 Equipe: Physiopathologie et Epidémiologie Cérébro-Cardiovasculaires (AE 7460, PEC2), UFR Sciences de Santé, Université de Bourgogne Franche-Comté, Dijon, France

## Abstract

Unbalanced nutrition early in life is increasingly recognized as an important factor in the development of chronic, non-communicable diseases at adulthood, including metabolic diseases. We aimed to determine whether transient postnatal overfeeding (OF) leads to liver stress-induced premature senescence (SIPS) of hepatocytes in association with liver structure and hepatic function alterations. Litters sizes of male C57BL/6 mice were adjusted to 9 pups (normal feeding, NF) or reduced to 3 pups during the lactation period to induce transient postnatal OF. Compared to the NF group, seven-month-old adult mice transiently overfed during the postnatal period were overweight and developed glucose intolerance and insulin resistance. Their livers showed microsteatosis and fibrosis, while hepatic insulin signaling and glucose transporter protein expressions were altered. Increased hepatic oxidative stress (OS) was observed, with increased superoxide anion production, glucose-6-phosphate dehydrogenase protein expression, oxidative DNA damage and decreased levels of antioxidant defense markers, such as superoxide dismutase and catalase proteins. Hepatocyte senescence was characterized by increased p21^WAF^, p53, Acp53, p16^INK4a^ and decreased pRb/Rb and Sirtuin-1 (SIRT-1) protein expression levels. Transient postnatal OF induces liver OS at adulthood, associated with hepatocyte SIPS and alterations in liver structure and hepatic functions, which could be mediated by a SIRT-1 deficiency.

## Introduction

The prevalence of overweight and obesity, which are important risk factors for cardiovascular and metabolic diseases such as type 2 diabetes, insulin resistance and non-alcoholic fatty liver disease^1,2^, has dramatically increased over recent decades. Particularly alarming is the large increase in childhood obesity worldwide over the past three decades^3^, associated with an increased occurrence of metabolic syndrome in children and adolescents as well as type-2 diabetes in young adults^4^. Results from epidemiological studies have shown that an altered nutritional environment during the critical developmental period early in life increases the risk of developing cardiovascular and metabolic disorders thereafter^5^. Notably, in humans, overnutrition during the perinatal period has been linked to obesity and the associated comorbidities. In rodents, reducing the litter size after birth decreases competition for milk during the suckling period and therefore induces over-nourishment in the postnatal period, which in turn leads to increased body weight and fat content at weaning^6^. Furthermore, transient postnatal overfeeding (OF) provokes metabolic (e.g., hyperphagia, overweight, insulin resistance, higher total and visceral fat mass, altered lipid profile) and cardiovascular (e.g., hypertension, cardiac dysfunction) disorders in adulthood, often associated with increased oxidative stress (OS)^7–15^. The liver is considered the most vulnerable organ to altered nutritional programming during the perinatal period^16^. The liver plays an important role in the maintenance of lipid and glucose homeostasis and is particularly susceptible to OS-induced damage^17,18^. The overproduction of reactive oxygen species (ROS) and nitrogen species can induce hepatocyte dysfunction, which contributes to the pathogenesis of acute and chronic liver diseases^19^ and to the onset or maintenance of glucose intolerance and insulin resistance^20^. Cell senescence, a sustained anti-proliferative response arresting the cell cycle, has also been associated with metabolic disorders^21^. Depending on the activating signals, senescence can be either replicative or premature. Replicative senescence is characterized by a finite replicative potential that limits the lifespan of a cell to a certain number of divisions. This process is induced *via* signals triggered by telomere shortening^22^. On the other hand, stress-induced premature senescence (SIPS) is caused in young cells *via* different mechanisms, such as OS^23^. Hepatocyte senescence has been linked to chronic liver disease^24^. However, to our knowledge, no studies have demonstrated the role of OS in association with adulthood hepatocyte SIPS and dysfunction secondary to early postnatal OF. Using a mouse model of transient postnatal OF induced by reducing the litter size during the lactation period, we studied the association between OS, SIPS, and liver structure and function alterations in adulthood. We also investigated whether such dysfunctions were already present earlier in life.

## Results

### Body weight and body composition

During the neonatal suckling period, weight gain was significantly higher in pups raised in OF mice. At postnatal day (PND) 24, the body weight of the OF litter was 63% higher than that of mice in the normal feeding (NF) litter (OF (n = 8) *vs*. NF (n = 8) (mean (g) ± SD): 12.65 ± 0.4 *vs*. 7.77 ± 0.46; p < 0.001) (Table 1). This difference persisted during growth and maturation, but to a lesser extent; at 7 months of life, the body weight in the OF group was 11.7% greater than that in the NF group (OF (n = 8) *vs*. NF (n = 8) (mean (g) ± SD): 34.69 ± 1.99 g *vs*. 31.05 ± 1.71 g; p < 0.001) (Table 1). However, a major increase in body fat mass (OF (n = 8) *vs*. NF (n = 8) (mean (%) ± SD): 14.9 ± 4.6 *vs*. 6.4 ± 1.1; p < 0.001) was observed in the OF group (Table 1).

**Table 1:** Body weight (g) and fat mass percentage of NF and OF mice at PND24 and 7 months of age.

	Postnatally normally fed (NF) mice; n = 8	Postnatally overfed (OF) mice; n = 8	Significance
Body weight (PND 24)	7.77 ± 0.46	12.65 ± 0.4	***
Body weight (7 months)	31.05 ± 1.71	34.69 ± 1.99	***
Fat mass percentage (7 months)	6.4 ± 1.1	14.9 ± 4.6	***

The values are reported as the mean ± SD; ***p < 0.001; n = 8 animals/group.

### Oxidative stress in hepatic tissues

**Figure 1:**
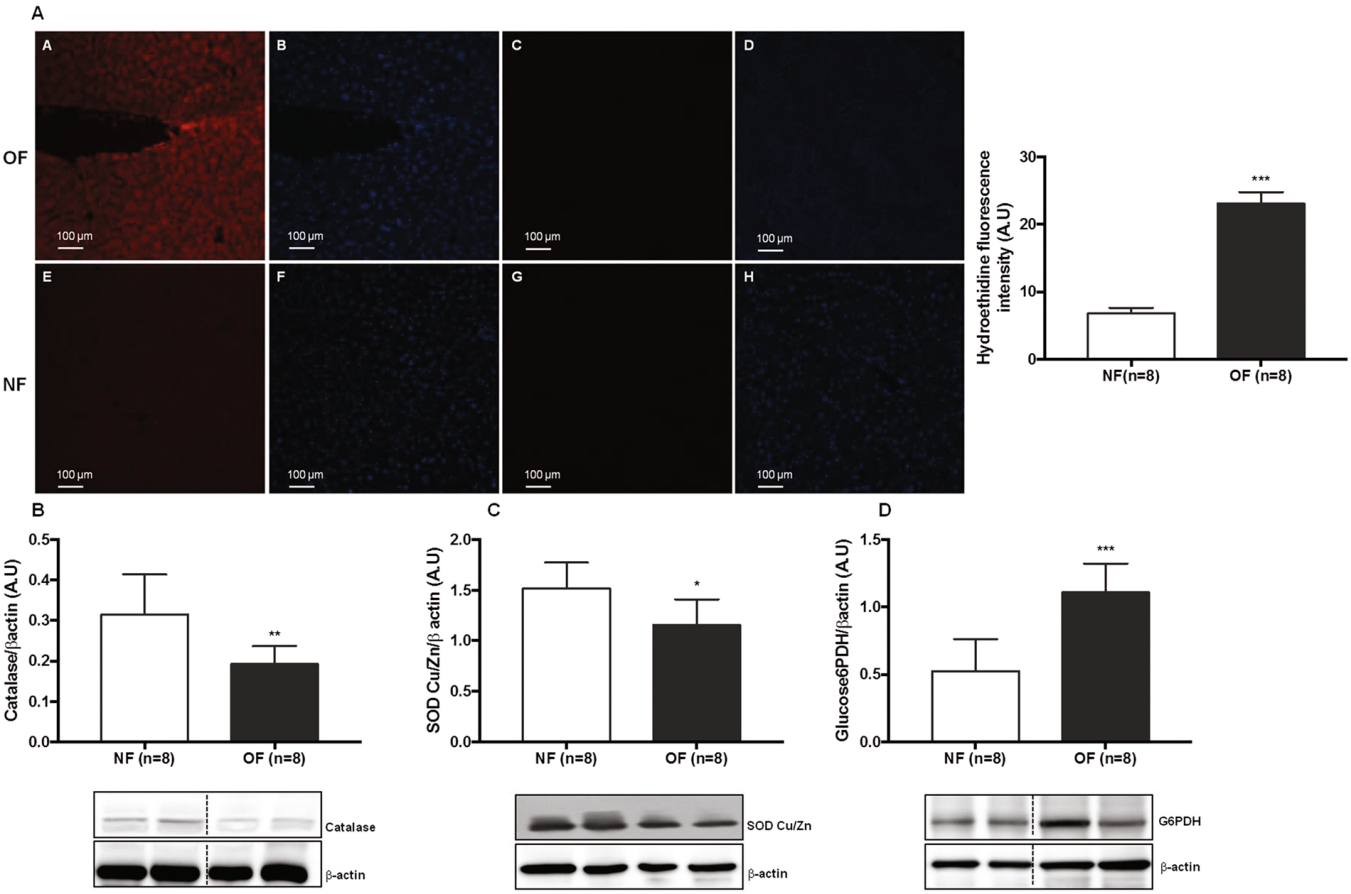
Hepatic oxidative stress in the NF and OF groups at 7 months of life. Superoxide anion production was measured by hydroethidine staining (**A**) at the same magnification (20x) in the OF (A-A) and NF (A-E) groups or without hydroethidine in the OF (A-C) and NF (A-G) groups. Nuclei were counterstained with DAPI (blue) (A-B, A-D, A-F, A-H). Hydroethidine fluorescence intensity was quantified using ImageJ (NF (white) and OF (grey)). Hepatic protein levels of CAT (**B**), SOD Cu/Zn (**C**) and G6PDH (**D**) were measured by western blot in the NF (white) and OF (gray) groups. Cropped blots are displayed. Representative images are presented and full-length western blots are presented in supplemental data 1. The values are reported as the mean ± SD; *p < 0.05; **p < 0.01; ***p < 0.001; n = 8 animals/group.

OS has been evaluated by superoxide anions levels (O_2_^•−^), DNA damage and protein expression of antioxidant defenses. Livers from the OF group compared with those from the NF group at 7 months of life showed a significantly increased O_2_^•−^ production as determined by chemiluminescence (OF *vs*. NF (A.U. ± SD) 23.09 ± 0.60 *vs*. 6.82 ± 0.28; p < 0.001) (Fig. 1A) and decreased catalase (CAT) (−39%; p < 0.01) (Fig. 1B) and superoxide dismutase Cu/Zn (SOD Cu/Zn) (−23%; p < 0.05) (Fig. 1C) levels. Glucose-6 phosphate dehydrogenase (G6PDH) protein expression was highly increased (+140%; p < 0.001) (Fig. 1D).

**Figure 2:**
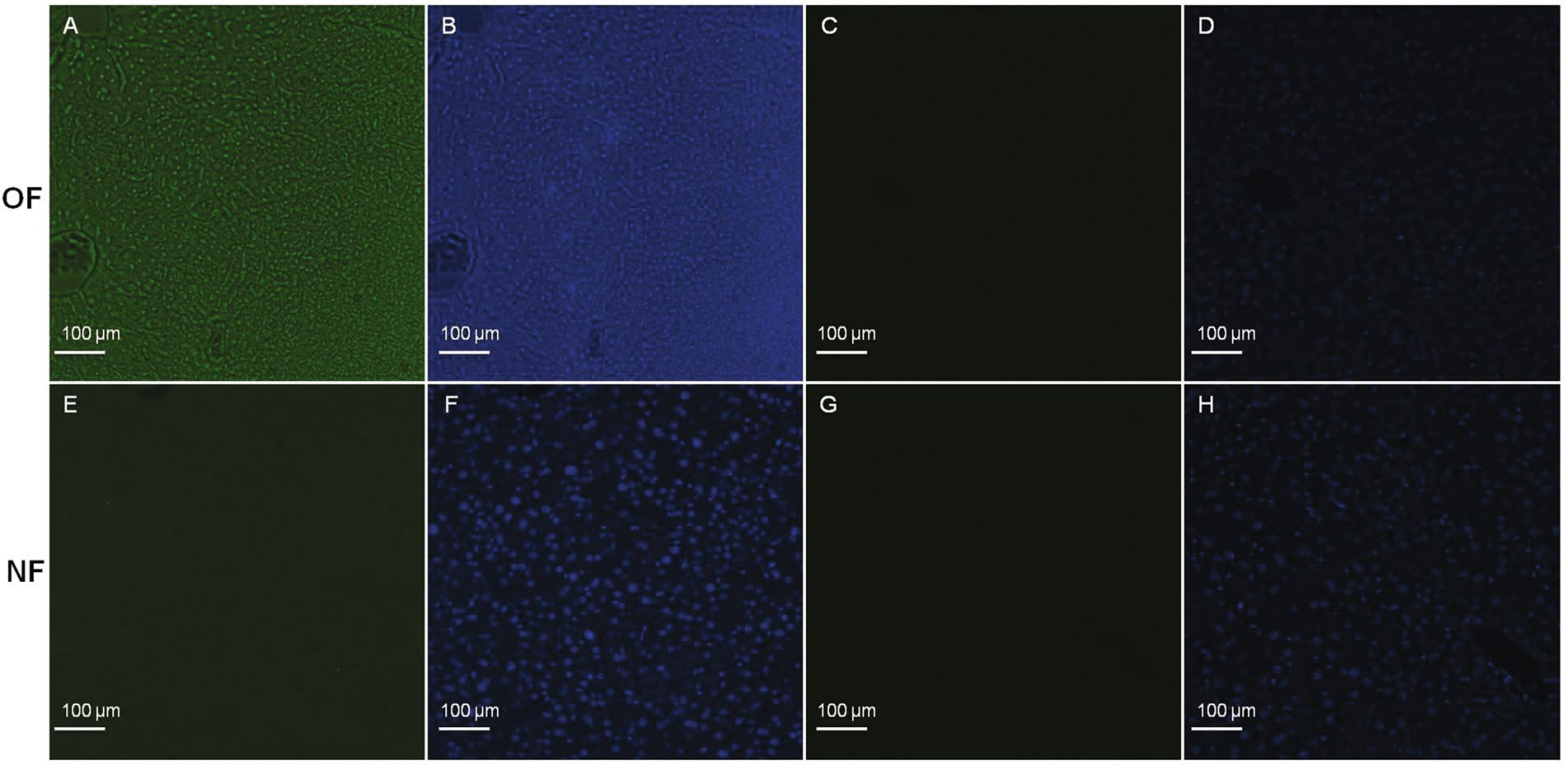
Hepatic oxidative DNA damage in the NF and OF groups at 7 months. Oxidative DNA damage was evaluated using 53BP-1 staining at the same magnification (20x) in the OF (**A**) and NF (**E**) groups. Nuclei were counterstained with DAPI (blue) (**B** and **F**). Negative control was included in OF (**C**) and NF (**G**) groups, and nuclei were counterstained with DAPI (**D** and **H**). These pictures are representative images from n = 8 animals/group.

Oxidative DNA damage was evaluated by chemiluminescence using 53BP-1 staining in adult animals (Fig. 2). Livers from the OF group compared with those from the NF group showed increased DNA damage as characterized by increased 53BP-1 staining (OF *vs*. NF (A.U. ± SD): 19.20 ± 0.26 *vs*. 14.25 ± 0.16; p < 0.001) (Fig. 2A vs. E).

**Figure 3:**
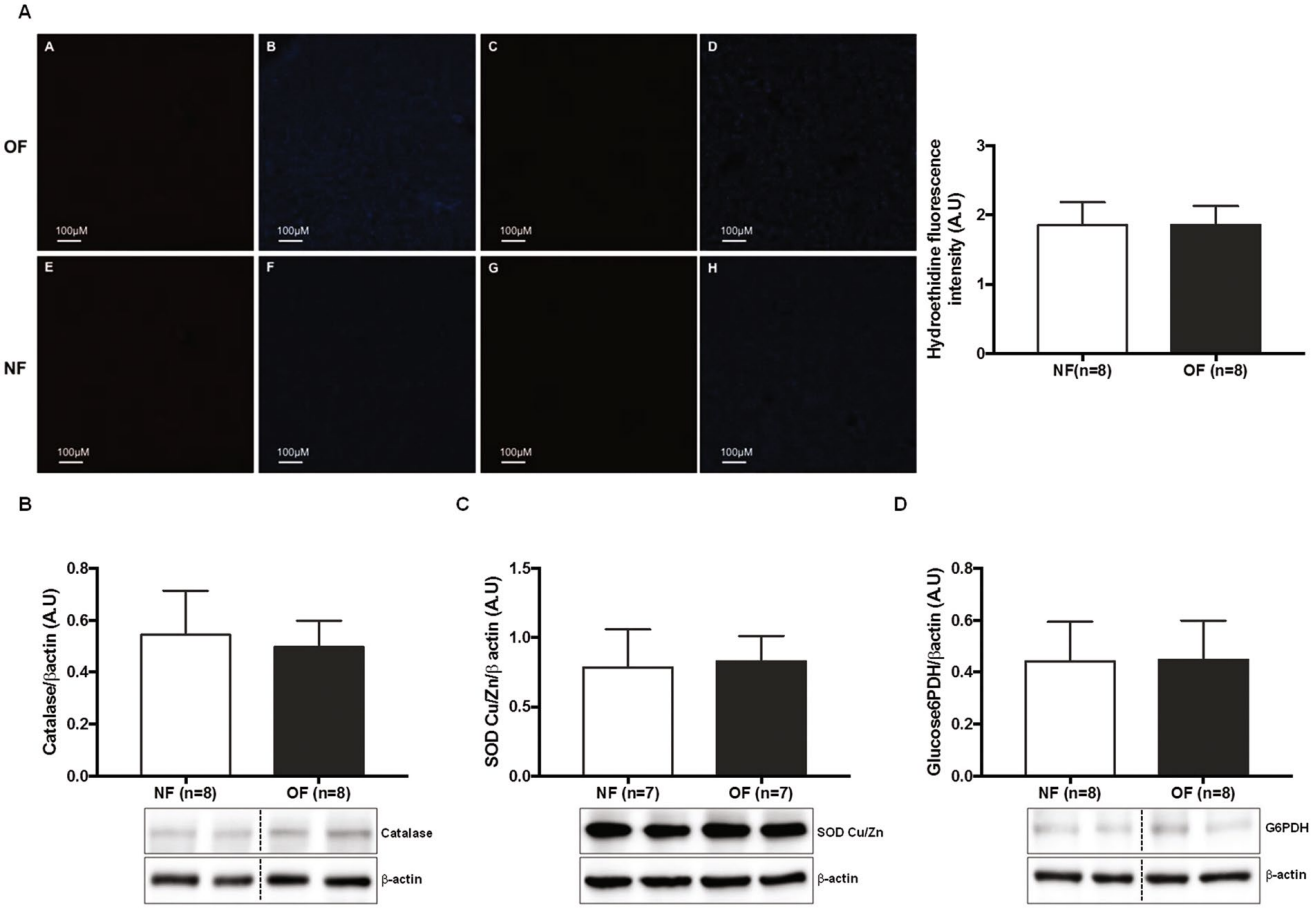
Hepatic oxidative stress in the NF and OF groups at PND 24. Superoxide anion production was measured by hydroethidine staining (**A**) at the same magnification (20x) in the OF (A-A) and NF (A-E) groups or without hydroethidine in the OF (A-C) and NF (A-G) groups. Nuclei were counterstained with DAPI (blue) (A-B, A-D, A-F, A-H). Hydroethidine fluorescence intensity was quantified using ImageJ (NF (white) and OF (gray)). These pictures are representative images from n = 8 animals/group. Hepatic protein levels of CAT (**B**), SOD Cu/Zn (**C**) and G6PDH (**D**) were measured by western blot in the NF (white) and OF (gray) groups. Cropped blots are displayed. Representative images are presented and full-length western blots are presented in supplemental data 2. The values are reported as the mean ± SD; p > 0.05; n = 7–8 animals/group.

At PND 24, O_2_^•−^ production in the liver was similar between the two groups (OF *vs*. NF (A.U. ± SD): 1.87 ± 0.09 *vs*. 1.85 ± 0.11; p > 0.05) (Fig. 3A). No difference in CAT (Fig. 3B), SOD Cu/Zn (Fig. 3C) or G6PDH (Fig. 3D) proteins expression was observed.

### Senescence in hepatic tissue

**Figure 4:**
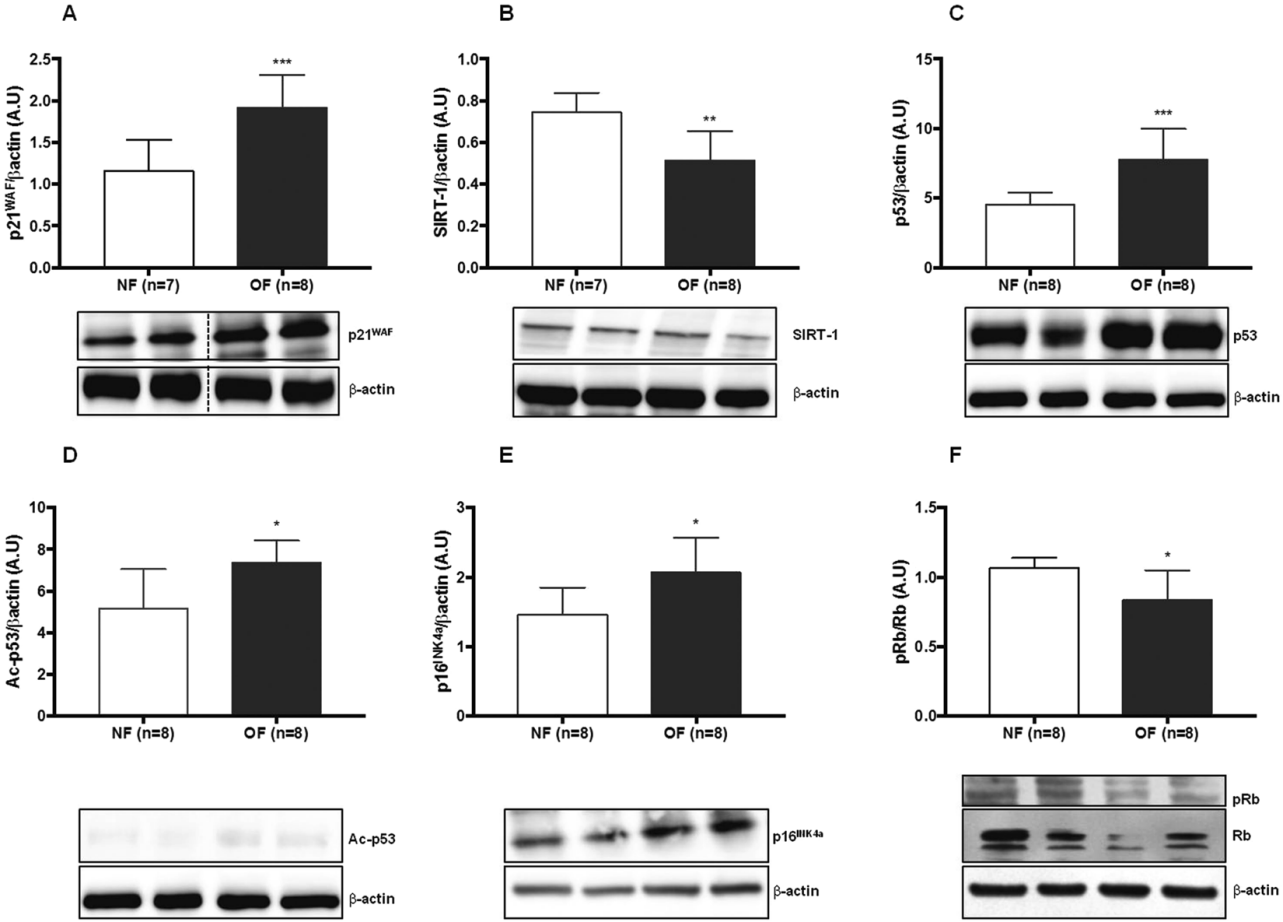
Hepatic senescence in the NF and OF groups at 7 months of life. Liver protein levels of p21^WAF^ (**A**), SIRT-1 (**B**), p53 (**C**), Ac-p53 (**D**), p16^INK4a^ (**E**) and pRb/Rb (**F**) were measured by western blot in the NF (white) and OF (gray) groups. Cropped blots are displayed. Representative images are presented and full-length western blots are presented in supplemental data 1. The values are reported as the mean ± SD; *p < 0.05; **p < 0.01; ***p < 0.001; n = 7–8 animals/group.

**Figure 5:**
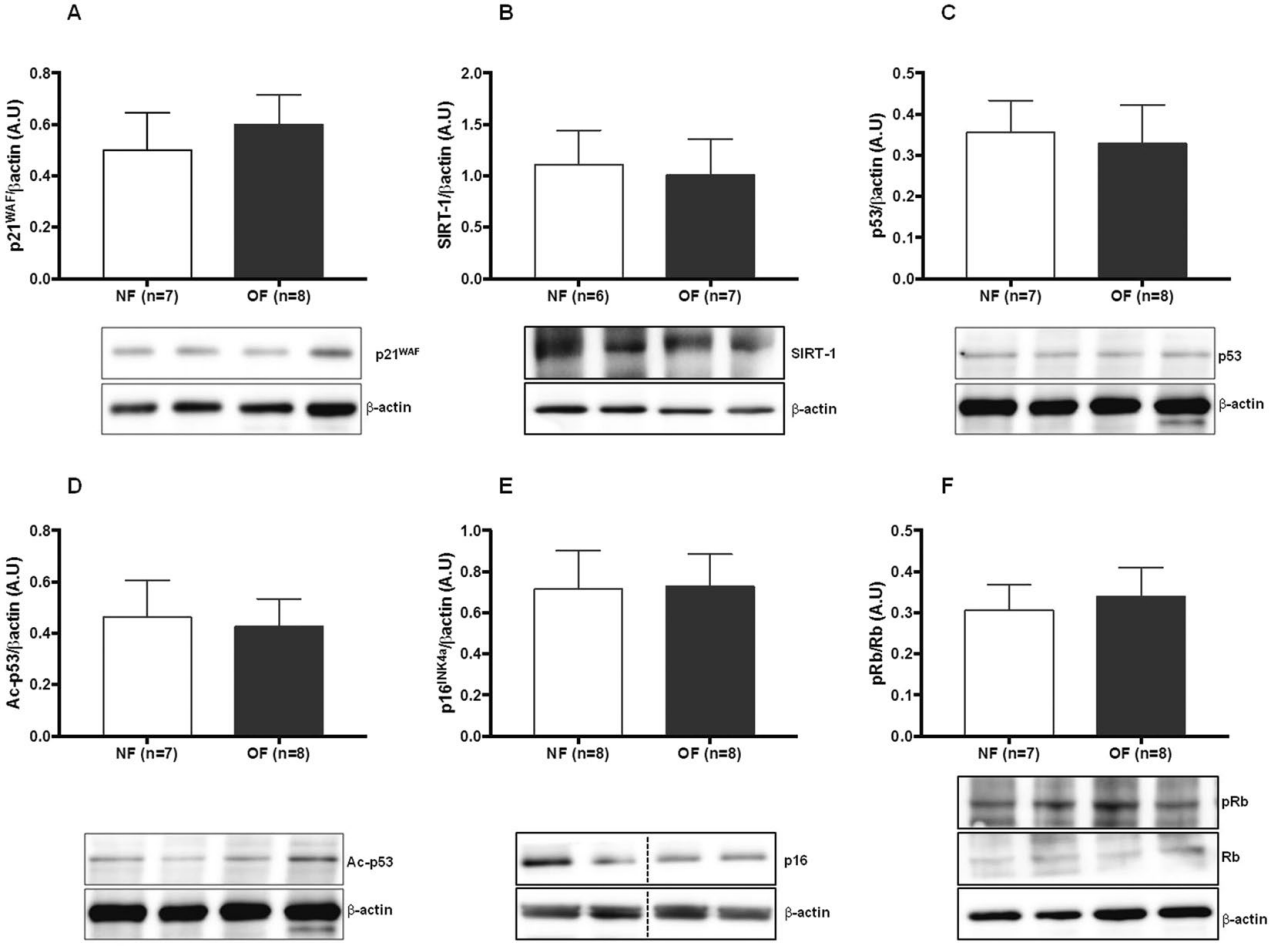
Hepatic senescence in the NF and OF groups at PND 24. Liver protein levels of p21^WAF^ (**A**), SIRT-1 (**B**), p53 (**C**), Ac-p53 (**D**), p16^INK4a^ (**E**) and pRb/Rb (**F**) were measured by western blot in the NF (white) and OF (gray) groups. Cropped blots are displayed. Representative images are presented and full-length western blots are presented in supplemental data 2. The values are reported as the mean ± SD; p > 0.05; n = 6–8 animals/group.

Hepatic senescence was investigated *via* western blot by measuring the protein expression of p21^WAF^, p53, Ac-p53, p16 ^INK4a^, p-Rb/Rb and SIRT-1, which are considered senescence markers. At 7 months of life, livers from the OF group compared with those from the NF group showed increased levels of p21^WAF^ (+60%; p < 0.001) (Fig. 4A), p53 (+60%; p < 0.001) (Fig. 4C), Acp53 (+43%; p < 0.05) (Fig. 4D), and p16^INK4a^ (+41%; p < 0.05) (Fig. 4E) expression. In contrast, the protein expression of pRb/Rb, (−21%; p < 0.05) (Fig. 4F) and SIRT-1 (−31%; p < 0.01) (Fig. 4B) was decreased. No differences were observed between the OF and NF groups at PND 24 in expression levels of p21^WAF^ (Fig. 5A), SIRT-1 (Fig. 5B), p53 (Fig. 5C), Acp53 (Fig. 5D), p16^INK4a^ (Fig. 5E), and pRb/Rb (Fig. 5F).

### Liver structure

**Figure 6:**
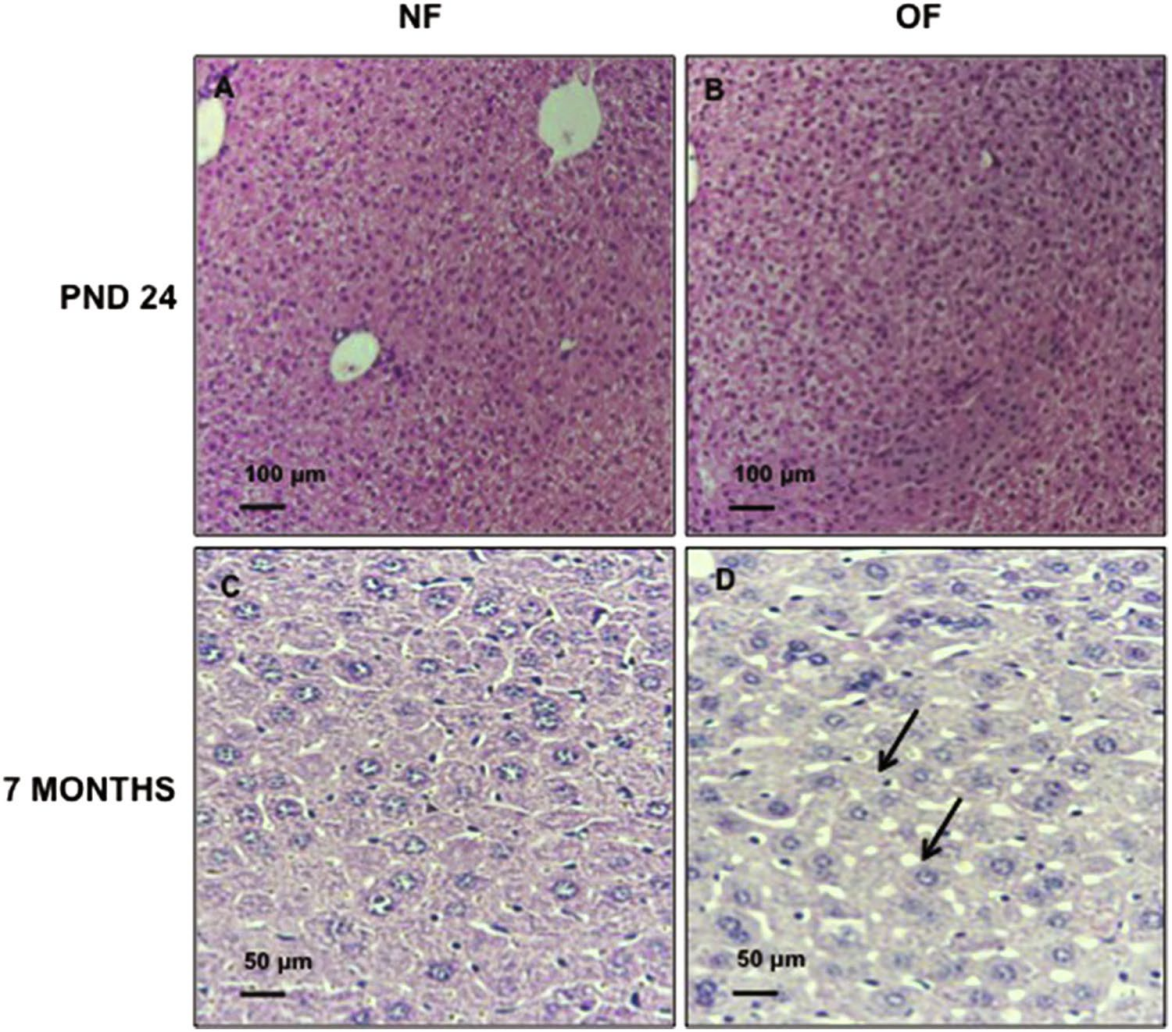
Hematoxylin and eosin staining in liver from the NF and OF groups at PND 24 and 7 months of life. Basic liver histology was observed by staining with hematoxylin (nuclear localization) and eosin (cytoplasmic localization) in the NF (**A**) and OF (**B**) groups at 20x at PND 24 and in the NF (**C**) and OF (**D**) groups at 40x at 7 months of age. Slight microsteatosis was observed (arrow) only at 7 months of life in OF (D) *vs*. NF (**C**) groups. These images are representative from n = 8 animals/group.

**Figure 7:**
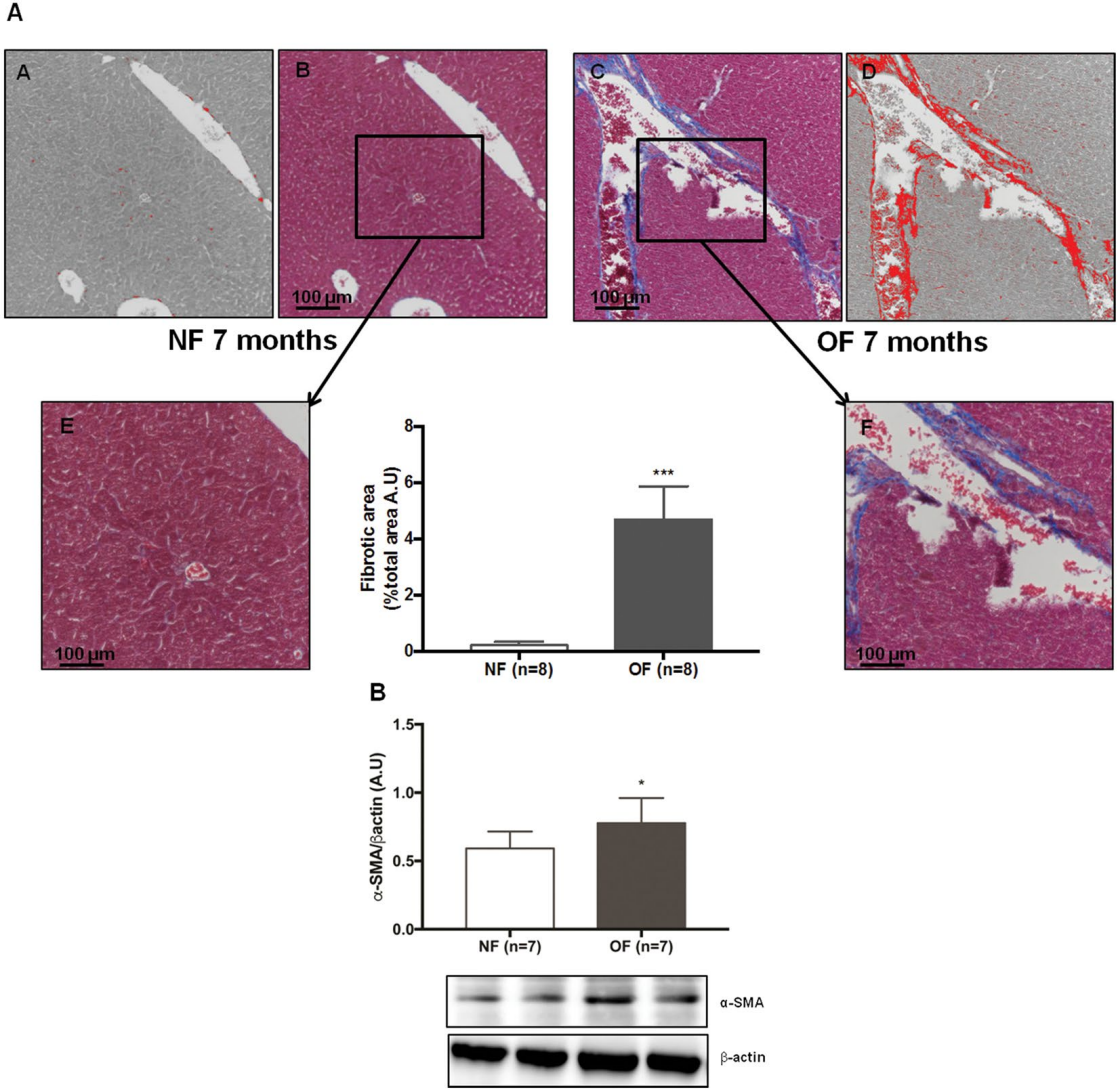
Hepatic fibrosis in the NF and OF groups at 7 months of life. Hepatic fibrosis was evaluated with Masson’s Trichrome at 10x in the NF (A-B) and OF (A-C) groups. A “stack image” was generated from NF (A-B) and OF (A-C) groups, respectively (A-A) and (A-D), to quantify the fibrotic area using ImageJ. A specific zone was enlarged at 20x in the NF (A-E) and OF (A-F) groups. ***p < 0.001. These images are representative from n = 8 animals/group. Alpha-SMA (α-SMA) protein expression was measured by western blot in the NF (white) and OF (gray) groups (**B**). Cropped blots are displayed. Representative images are presented and full-length western blots are presented in supplemental data 1. The values are reported as the mean ± SD; *p < 0.05; n = 7 animals/group.

**Figure 8:**
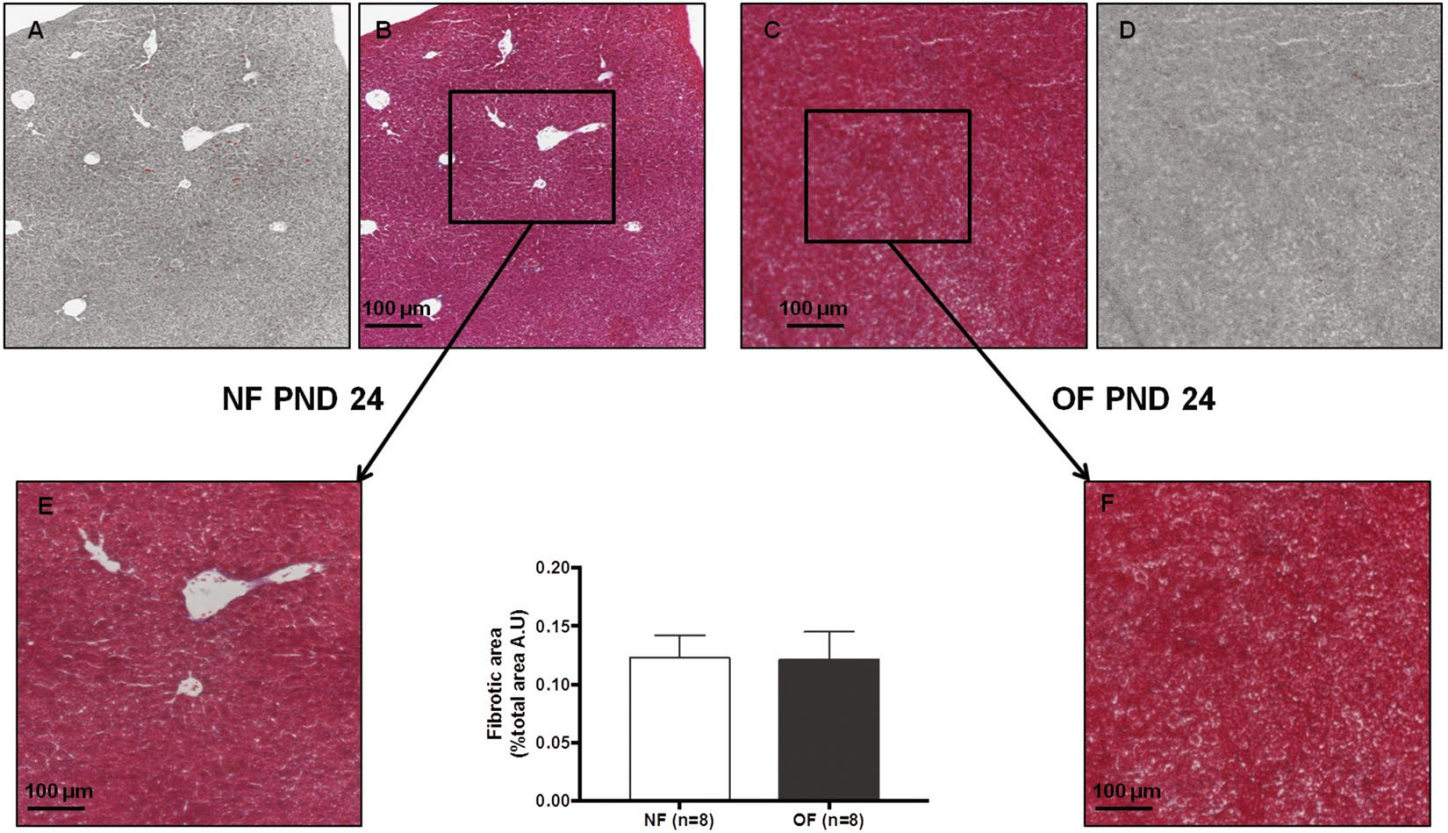
Hepatic fibrosis in the NF and OF groups at PND24. Hepatic fibrosis was evaluated with Masson’s Trichrome at 10x in the NF (**B**) and OF (**C**) groups. A “stack image” was generated from NF (**B**) and OF (**C**) groups, respectively (**A**) and (**D**), to quantify the fibrotic area using ImageJ. A specific zone was enlarged to 20x in the NF (**E**) and OF (**F**) groups. These images are representative from n = 8 animals/group. p > 0.05.

Liver structure has been evaluated using histological staining (Figs 6, 7, 8) at PND 24 and 7 months of life. By 7 months of life, hepatic structure the OF group was suggestive of dysfunction. Livers from the OF group compared with those from the NF group exhibited a mild to moderate microsteatosis (Fig. 6D *vs*. C) upon hematoxylin and eosin (H&E) staining. Hepatic fibrosis was identified using Masson’s Trichrome staining in livers from the OF group compared with those from the NF group (Fig. 7A,C *vs*. B) and fibrosis was quantified using ImageJ. We observed increased fibrotic areas in the OF group compared with the NF group (OF *vs*. NF (A.U. ± SD) 4.69 ± 0.41 *vs*. 0.22 ± 0.03; p < 0.001) (Fig. 7A,D *vs*. A). Hepatic fibrosis was confirmed by increased alpha-SMA (α-SMA) protein levels in livers from the OF group compared to the NF group (+30%, p < 0.05) (Fig. 7B). At PND 24, histological analyses did not show differences between the two groups (Figs 6A *vs*. B and 8).

### Histological detection of senescence

**Figure 9:**
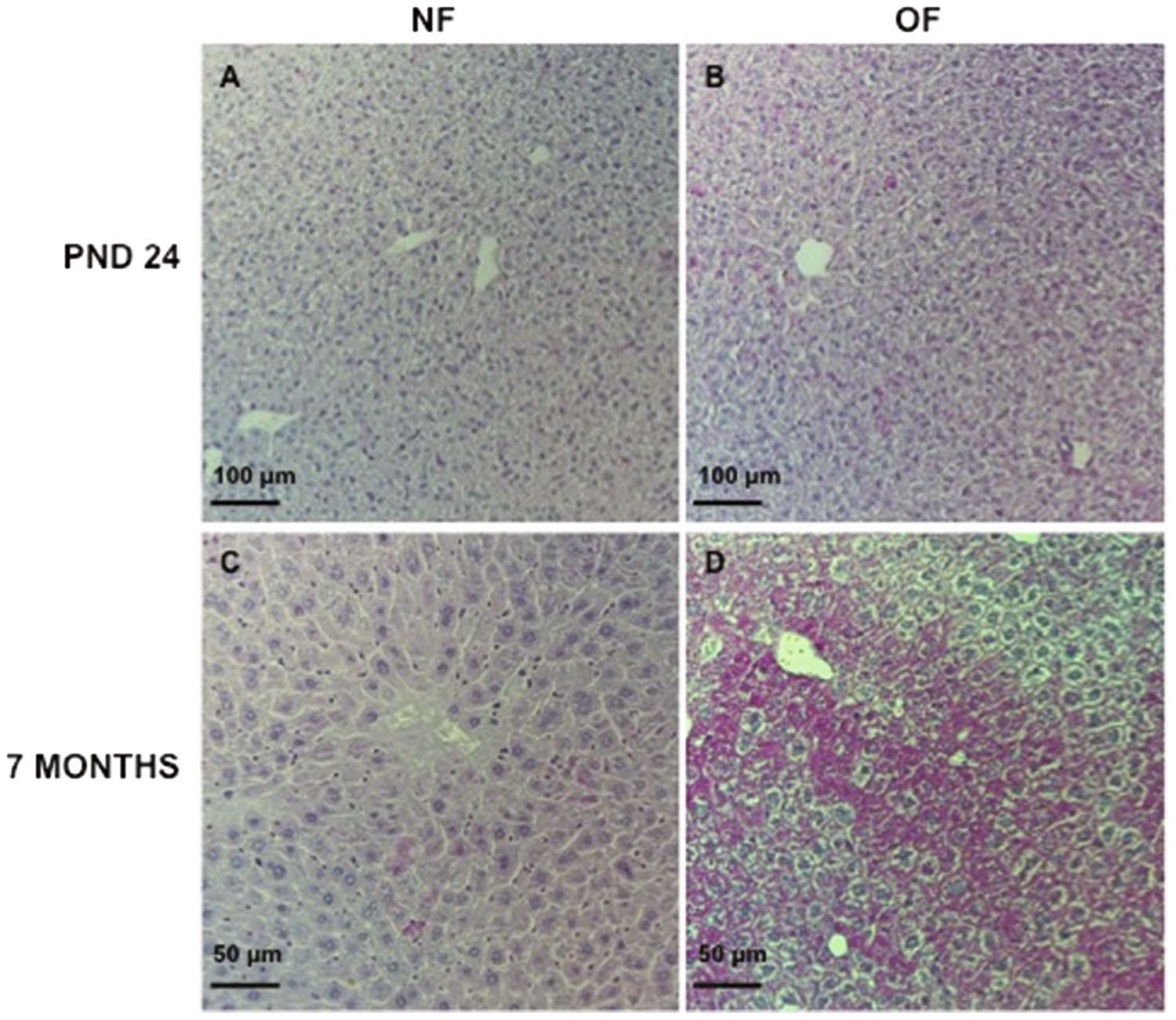
Diastase periodic acid-Schiff (d-PAS) staining in livers from the NF and OF groups at PND 24 and 7 months of life. Livers from the NF and OF groups were stained with d-PAS at PND 24 (**A** and **B**, respectively) (20x) and at 7 months of life (**C** and **D**, respectively) (40x). These images are representative from n = 8 animals/group.

**Figure 10:**
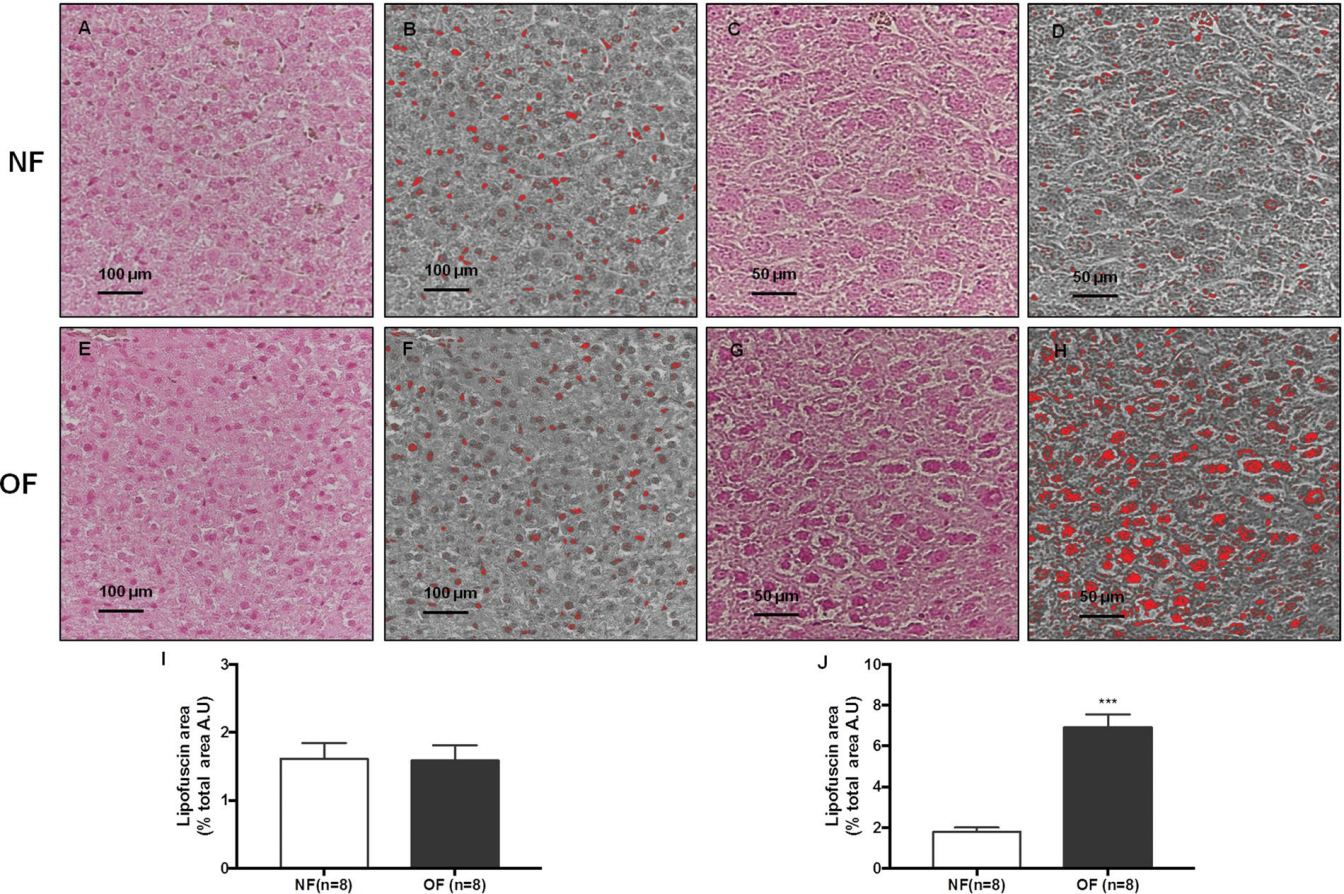
Lipofuscin staining with Fontana-Masson in liver from the NF and OF groups at PND 24 and 7 months of life. Lipofuscin staining was evaluated with Fontana-Masson at PND 24 in the NF (**A**) and OF (**E**) groups (20x) and at 7 months of life in the NF (**C**) and OF (**G**) groups (40x). A “stack image” was generated from NF (**B**) and OF (**F**) groups at PND 24 and from NF (**D**) and OF (**H**) groups at 7 months of life to quantify the stained structure area using ImageJ. The results are reported as the percentage of lipofuscin staining area of the total area. ***p < 0.001. These images are representative from n = 8 animals/group.

**Figure 11:**
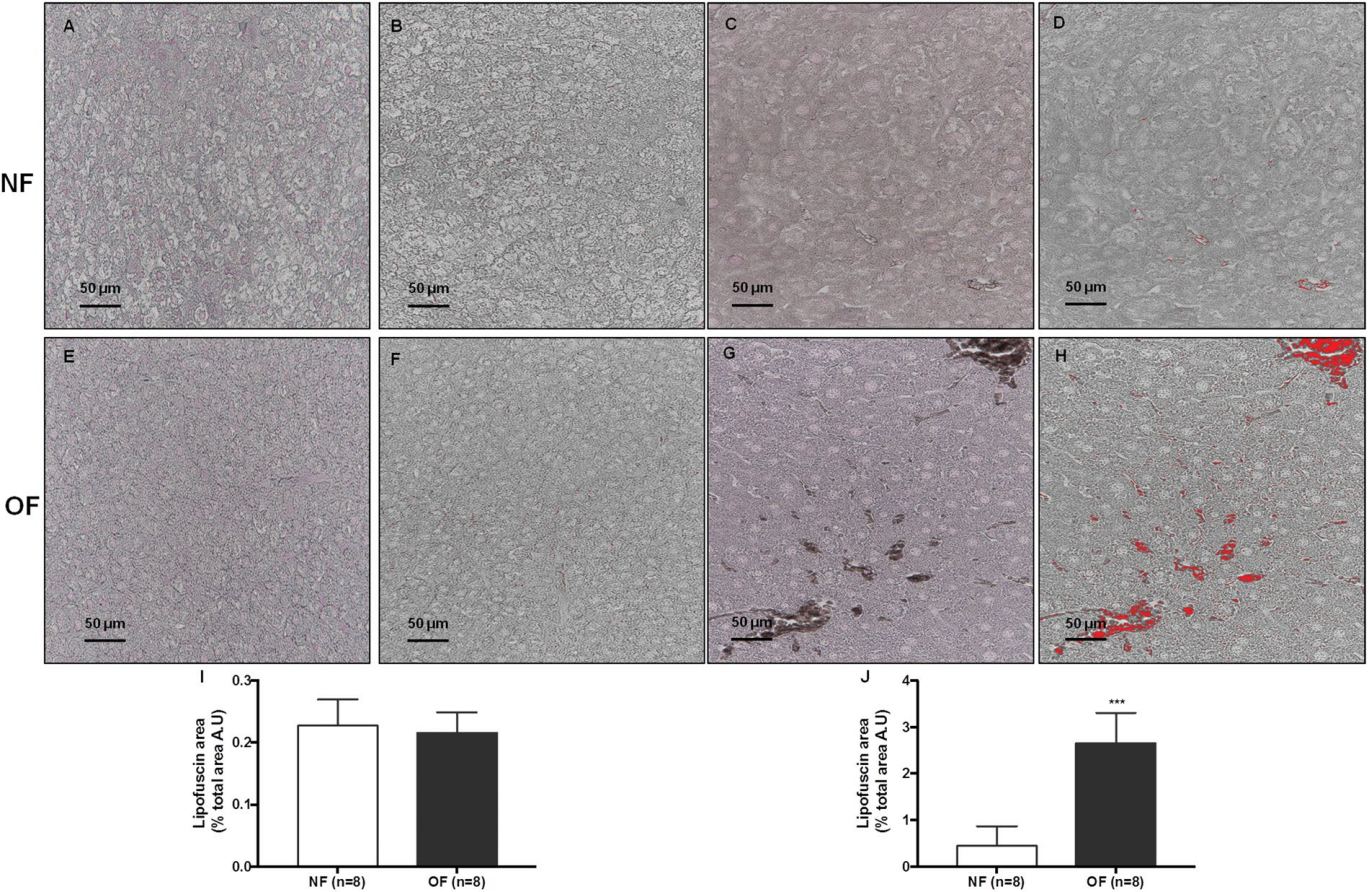
Lipofuscin staining with Sudan Black B in liver from the NF and OF groups at PND 24 and 7 months of life. Lipofuscin staining was evaluated with Sudan Black B at PND 24 in the NF (**A**) and OF (**E**) groups (40x) and at 7 months of life in the NF (**C**) and OF (**G**) groups (40x). A “stack image” was generated from NF (**B**) and OF (**F**) groups at PND 24 and from NF (**D**) and OF (**H**) groups at 7 months of life to quantify the stained structure area using ImageJ. The results are reported as the percentage of lipofuscin staining area of total area. ***p < 0.001. These images are representative from n = 8 animals/group.

At 7 months of life, the cytoplasmic accumulation of highly oxidized insoluble proteins, known as lipofuscin, was evaluated in livers from the OF group compared with those from the NF group. Such proteins are diastase periodic acid-Schiff (d-PAS) resistant (Fig. 9D *vs*. C). They were characterized by increased Fontana-Masson staining (OF *vs*. NF (A.U. ± SD) 6.92 ± 0.22 *vs*. 1.80 ± 0.06; p < 0.001) (Fig. 10H *vs*. D and J) and Sudan Black B (SBB) staining (OF *vs*. NF (A.U. ± SD) 2.72 ± 0.24 *vs*. 0.46 ± 0.16; p < 0.001) (Fig. 11H *vs*. D and J). At PND 24, we did not find differences between livers from NF and OF groups regarding d-PAS (Fig. 9B *vs*. A), Fontana-Masson (OF *vs*. NF (A.U. ± SD) 1.58 ± 0.08 *vs*. 1.61 ± 0.08; p > 0.05) (Fig. 10F *vs*. B and I) or SBB (OF *vs*. NF (A.U. ± SD) 0.21 ± 0.01 *vs*. 0.22 ± 0.01; p > 0.05) (Fig. 11F *vs*. B and I) staining.

### Glucose tolerance and insulin tolerance test (GTT and ITT)

**Figure 12:**
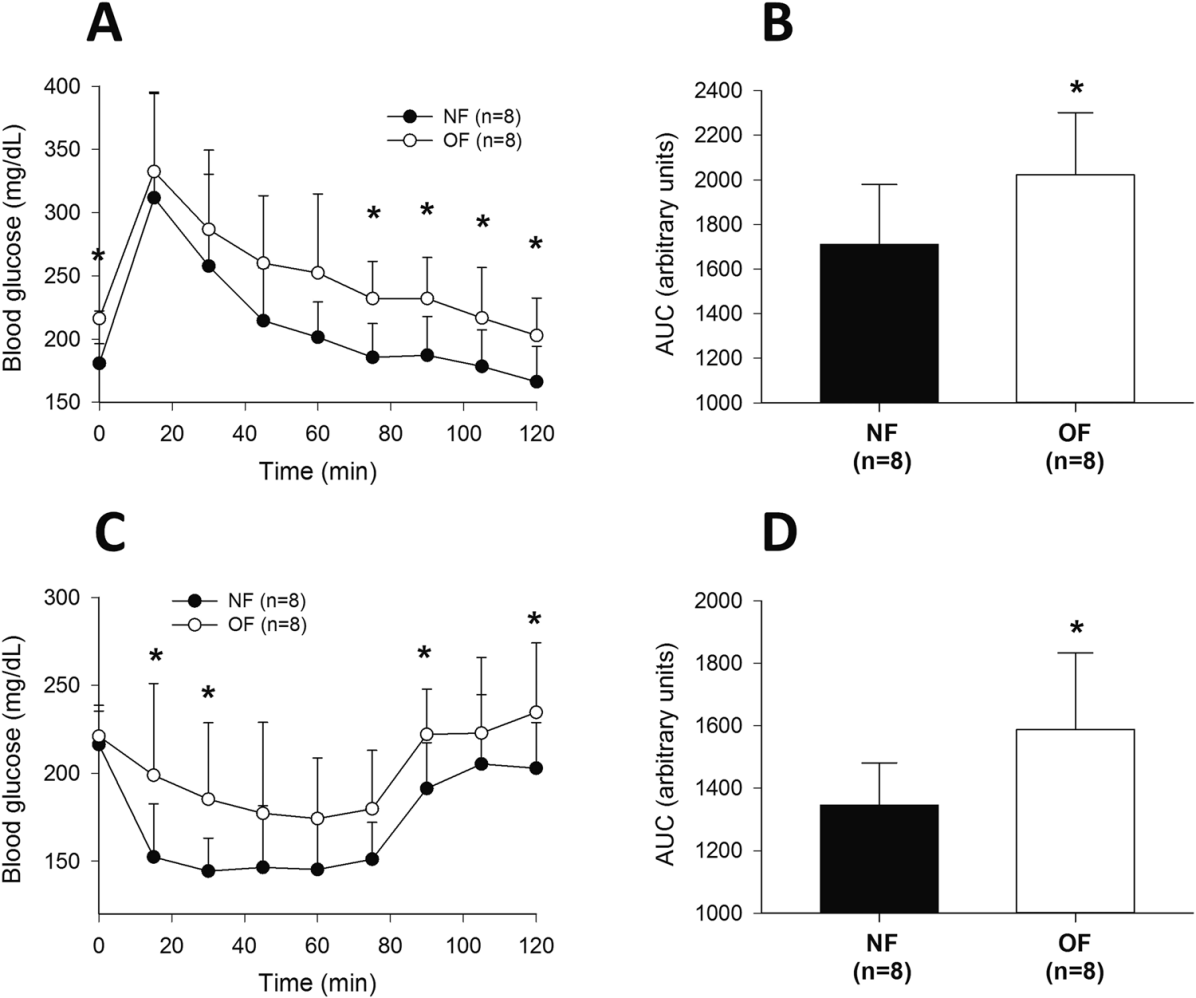
Intraperitoneal glucose and insulin tolerance tests at 6 months of age in the NF and OF groups. Evolution of blood glucose (**A**,**C**) and the area under the curve for blood glucose (**B**,**D**) after 2 g/kg glucose (**A**,**B**) or 0.75 IU/kg insulin (**C**,**D**) was intraperitoneally administered in the NF (black) and OF (white) groups. The values are reported as the mean ± SD; *p < 0.05; n = 8 animals/group.

To assess the glucose tolerance and insulin resistance of the OF and NF groups, intraperitoneal glucose and insulin tests were performed at 6 months of life. Blood glucose concentrations were higher in OF mice than in NF mice early after glucose injection (Fig. 12A) (p < 0.05), and the area under the curve (AUC) of the blood glucose concentration was significantly greater in the OF group than in the NF group (Fig. 12B) (p < 0.05), indicating altered tolerance to glucose in this group. The ITTs revealed a lower decrease in blood glucose in the OF group (Fig. 12C,D) (p < 0.05), corresponding to insulin resistance in this group.

### Insulin signaling in the liver

**Figure 13:**
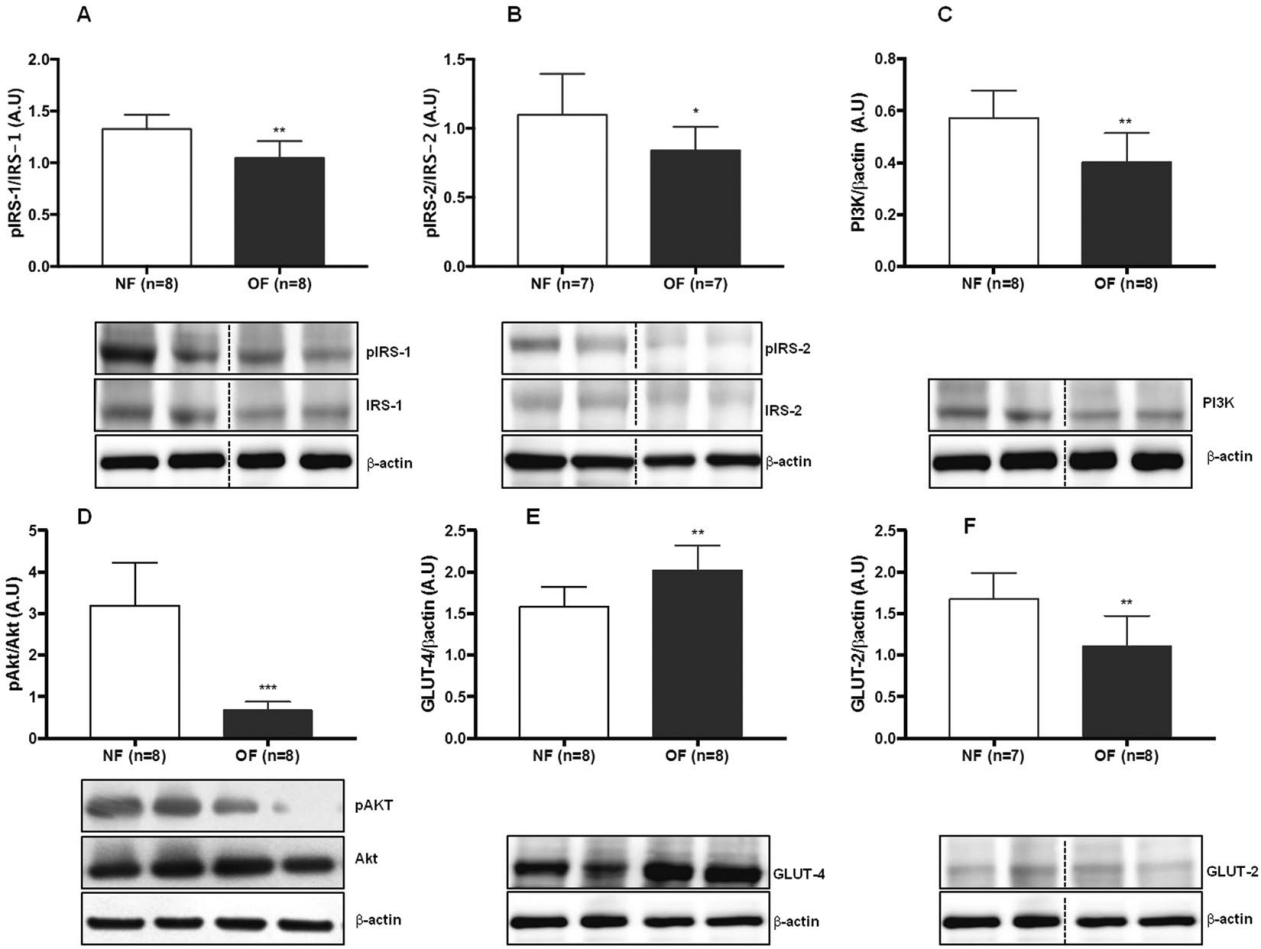
Liver insulin signaling and glucose transporter levels in the NF and OF groups at 7 months of life. Liver protein levels of pIRS-1/IRS-1 (**A**), pIRS-2/IRS-2 (**B**), PI3K (**C**), pAkt/Akt (**D**), GLUT-4 (**E**), GLUT-2 (**F**) were measured by western blot in the NF (white) and OF (gray) groups. Cropped blots are displayed. Representative images are presented and full-length western blots are presented in supplemental data 1. The values are reported as the mean ± SD; *p < 0.05; **p < 0.01; ***p < 0.001; n = 7–8 animals/group.

**Figure 14:**
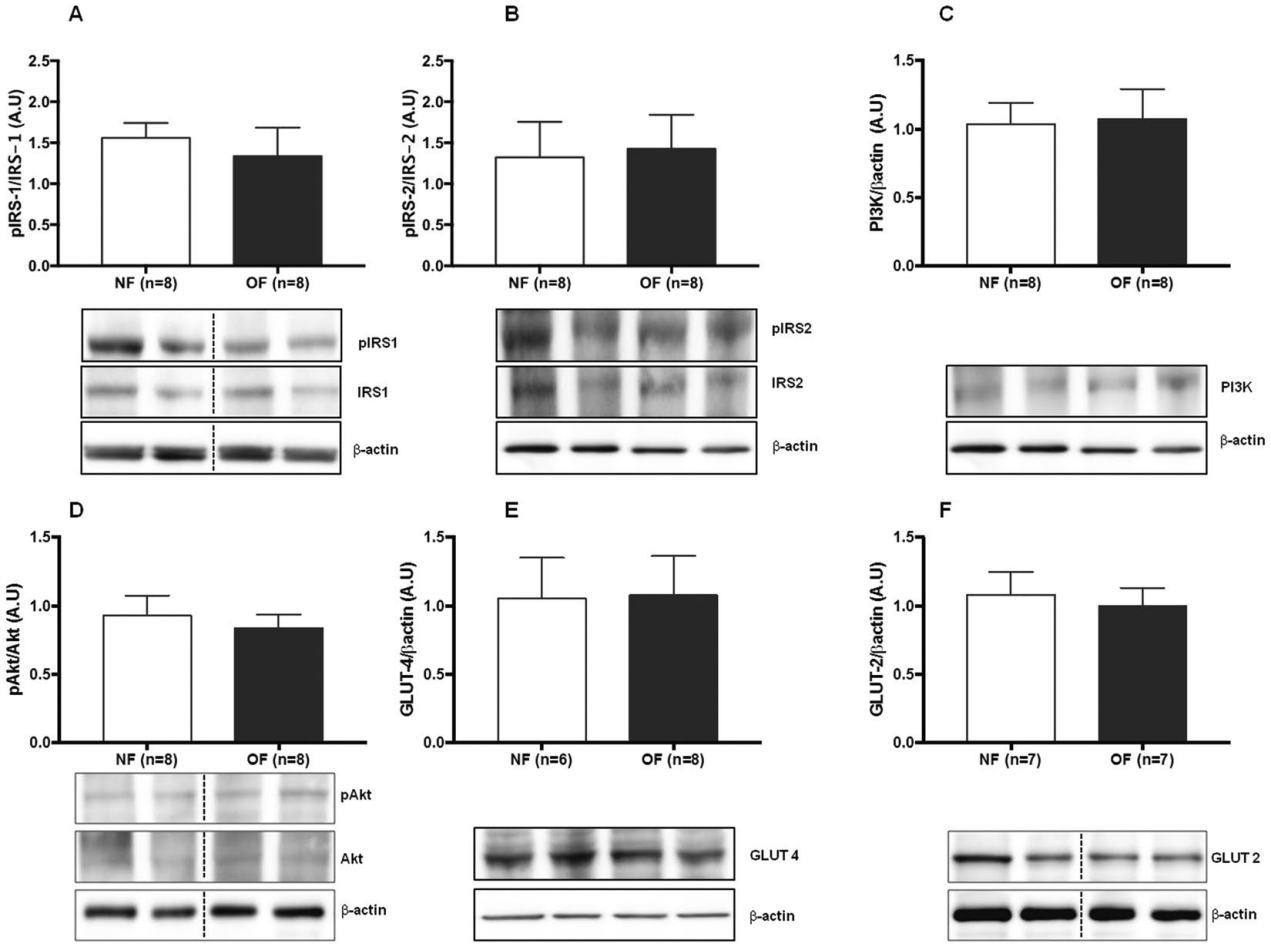
Liver insulin signaling and glucose transporter levels in the NF and OF groups at PND 24. Liver protein levels of pIRS-1/IRS-1 (**A**), pIRS-2/IRS-2 (**B**), PI3K (**C**), pAkt/Akt (**D**), GLUT-4 (**E**), GLUT-2 (**F**) were measured by western blot in the NF (white) and OF (gray) groups. Cropped blots are displayed. Representative images are presented and full-length western blots are presented in supplemental data 2. The values are reported as the mean ± SD; p > 0.05; n = 6–8 animals/group.

The expression levels of the insulin-signaling molecules pIRS-1/IRS-1, pIRS-2/IRS-2, Akt, PI3K, and pAkt/Akt were measured by western blot in livers from both the OF and NF groups. At 7 months of life, the livers from the OF group compared with those from the NF group showed decreased protein expression of pIRS-1/IRS-1 (−22%; p < 0.01) (Fig. 13A), pIRS-2/IRS-2 (−23%; p < 0.05) (Fig. 13B), PI3K (−25%; p < 0.01) (Fig. 13C), and pAkt/Akt (−74%; p < 0.001) (Fig. 13D). At PND 24, protein expression levels of pIRS-1/IRS-1 (Fig. 14A), pIRS-2/IRS-2 (Fig. 14B), PI3K (Fig. 14C), and pAkt/Akt (Fig. 14D) were similar between the two groups.

### Glucose transporters in the liver

The expression levels of the glucose transporters GLUT-4 and GLUT-2 were measured by western blot. At 7 months of life, livers from the OF group compared with those from the NF group showed decreased GLUT-2 protein expression (−30%; p < 0.01) (Fig. 13F), whereas GLUT-4 protein expression was increased (+27%; p < 0.01) (Fig. 13E). At PND 24, no differences in GLUT-2 (Fig. 14F) or GLUT-4 (Fig. 14E) proteins expression were observed between the two groups.

## Discussion

Clinical and experimental studies have shown that an altered nutritional environment during critical developmental periods contributes to the later development of metabolic disorders. Early exposure to suboptimal postnatal overnutrition, to maternal undernutrition or overnutrition, or to an imbalanced maternal diet can impair metabolic programming and therefore lead to the development of metabolic syndrome and related diseases at adulthood^16^. Many organs and systems have been identified as being sensitive to nutritional programming, notably, the liver. The liver is involved in protein, lipid and glucose homeostasis and therefore is particularly susceptible to OS-induced damage, including hepatocyte senescence, which has been linked to both acute and chronic liver diseases. However, the involved mechanisms remain unknown. In this study, using a transient postnatal OF mouse model induced by litter size reduction during the lactation period, we show that early excess nutrition during lactation period is associated with increased liver OS, accelerated hepatocyte senescence and altered hepatic structure and function.

We observed an increased OS in adult livers from the OF group compared with those from the NF group, characterized by increased O_2_^•−^ production and decreased CAT and SOD Cu/Zn protein expression levels as mentioned in other animal model of obesity^25^. Redox homeostasis is also regulated by G6PDH. In adipocytes, G6PDH overexpression has been shown to stimulate OS and promote the expression of pro-oxidative enzymes, mainly NADPH oxidase, which is responsible for the production of ROS and, notably, O_2_^•−^^26^. Livers from the OF group showed G6PDH overexpression at adulthood, a situation that has been observed in other animal models of obesity^27^ and which could therefore contribute to increase O_2_^•−^ production observed in this group. OS can induce DNA damage^28^. 53BP-1 is a protein involved in double-stranded break repair, and has been found to be elevated in many DNA damage response conditions^29^. We observed an increased 53BP-1 staining in livers from the OF group at 7 months of life, suggesting that transient postnatal OF appears to be associated with double-stranded DNA breaks in the liver induced by OS, which could contribute to the development of liver disease. Indeed, in patients with chronic liver disease, hepatic oxidative DNA damage, measuring by 8-hydroxy-2′-deoxyguanosine accumulation has been described^30^. This study shows the breaks induced by OS secondary to transient postnatal overnutrition. We investigated whether OS was also present earlier in life. At PND 24, we observed no differences between the OF and NF groups concerning liver O_2_^•−^ production and CAT, SOD Cu/Zn and G6PDH protein expression levels. These data are consistent with the latency period between the early stimulus and the later consequence, characteristic of altered developmental programming.

Excessive ROS levels and impaired DNA damage repair can contribute to premature cellular senescence and accelerated aging^31^ and are associated with metabolic and liver dysfunctions. We observed SIPS in livers from OF group, based on a multi-marker approach. Senescence being a multi-factorial process, and measurement of β-galactosidase (SA-β-gal) activity, the most extensively used biomarker of cellular senescence^32^, being impossible in the fixed preparations we used in the study, we chose to use the accumulation of lipofuscine as a marker of highly oxidized insoluble proteins and of senescence^33,34^ and found a lipofuscin accumulation in livers from 7-month-old OF mice. Lipofuscin staining has been proposed as a senescence biomarker, particularly for SIPS, comparable to SA-β-gal activity^35^, and has been associated with chronic OS^34^. We observed at adulthood increased p53, p21^WAF^, and p16^INK4a^ expression and reduced pRb/Rb expression as observed in chronic liver disease^36^. Transactivation of the p53 pathway^37^ and the cyclin-dependent kinase inhibitors p21^WAF^ and p16^INK4a^ ^38^ inhibits cyclin-dependent kinases, thereby preventing the phosphorylation of pRb, resulting in the silencing of genes involved in proliferation. An increased p16^INK4a^ protein expression could be linked to higher O_2_^•−^ levels observed in OF group. Indeed, upregulated p16^INK4a^ protein level has been observed in melanocytes treated with ROS (hydrogen peroxide), and this upregulation was blocked by pretreatment with the antioxidant *N*-acetylcysteine^39^, thus confirming the association between p16^INK4a^ upregulation and OS. Furthermore, the activation of p53 and p16^INK4a^ signaling has been reported in aged vessels, the visceral fat of obese individuals, as well as in aging rodent models, which could contribute to the progression of cardiovascular and metabolic disorders^40–42^.

The control of cellular senescence involves another family of proteins called sirtuins^43^. Sirtuins, that are collectively referred as deacylases, are a group of seven highly conserved proteins (SIRT-1 to SIRT-7) controlling the dynamic structure and function of chromatin; these proteins are divided into three classes based on homology with the yeast proteins Rpd3 (class I), Hda1 (class II) and Sir 2 (class III)^44^. Sir2 promotes the increase in life span induced by caloric restriction. SIRT-1 is the closest homolog to Sir2 and the best understood in terms of cellular activity and function, particularly in the regulation of cellular lifespan. An overexpression of SIRT-1 alone^45^ or the co-overexpression of SIRT-1 with nicotinamide phosphoribosyltransferase^46^ has been shown to delay cellular senescence, whereas inhibiting SIRT-1 expression and/or activity leads to premature cellular senescence^47^. Livers from the OF group showed a decreased SIRT-1 expression at adulthood compared with those from the NF group. Acetylation of p53 at Lys-382, a target of SIRT-1, was significantly higher in the OF group, suggesting an overall reduction in SIRT-1 activity^48^. Similar observations have been made in adipose tissue from OF mice^49^ and in primary cultures of senescent mouse hepatocytes^50^. OS, and notably ROS, can modulate SIRT-1 expression and activity^51^. Therefore, an increased superoxide anion production observed in OF group could explain the reduced SIRT-1 expression and activity observed in this group. Increased p16^INK4a^ expression with reduced Rb phosphorylation blocking the entry of proliferating cells into the S phase and decreased SIRT-1 protein expression and activity indicate that transient postnatal OF is associated with adult SIPS rather than with replicative senescence^48^. We also searched for signs of senescence earlier in life. At PND 24, no differences were observed in lipofuscin staining as well as in the protein levels of p53, Acp53, p21, p16^INK4a^, pRb/Rb and SIRT-1 between the OF and NF groups, indicating the absence of SIPS at an age where OS was absent, confirming the association between OS and SIPS.

On the other hand, this study demonstrates that transient, spontaneous OF during lactation period led to increased body weight at weaning (PND 24), which persisted to a lesser extent to adulthood, and a major increase in fat mass. Hepatic steatosis is an important structural alteration associated with obesity^52^. Obesity increases lipid delivery to the liver from both the diet and adipose tissue^53^. We observed the presence of a mild microsteatosis in livers from the OF group compared with those from the NF group only at adulthood, which could be the consequence of OS, notably decreased SOD and CAT expression levels as observed in a rat model of early postnatal overnutrition^25^, and also due to the higher percentage of fat mass observed in this group. Liver steatosis can be associated with OS caused by the lipid overload^54^ and decreased antioxidant enzymes activity^25,55^. Hepatic microsteatosis and macrosteatosis can be inter-converted^56^. At the time points in our study, we observed only a mild microsteatosis at 7-month-old OF mice and we can only speculate about a possible later transition to macrosteatosis with increasing age. Liver steatosis is an important factor that may determine the rate of fibrosis progression in a range of liver diseases. Hepatocytes from the OF group, compared with those from the NF group, showed an hepatic fibrosis at adulthood characterized by increased Masson’s Trichrome staining and alpha-SMA protein expression, a useful marker of the earliest stages of hepatic fibrosis^57^. Increased liver fibrosis could be a consequence of increased p53, p16^INK4a^ expression^58^ and decreased SIRT-1 activity/ expression^59^ that we observed in OF group.

Transient, spontaneous OF during the lactation period has been also associated with the development of subsequent metabolic disorders at adulthood. We observed in OF group at 7 months of age an impaired glucose tolerance and insulin resistance. Similar data have been reported in young OF mice at weaning^60^, and in adult OF rats^6,14^. Aging in humans and in animal studies has also been associated with the loss of insulin sensitivity^61^. Insulin resistance in liver has been associated with reduced expression of both major IRS-1 and IRS-2 proteins^62^, and with alteration in signaling through the IRS proteins, which coordinate various signals regulating cell growth, survival, and metabolism, notably *via* activation of the PI3K-Akt cascade^63^. We observed, only at adulthood, that livers from the OF group compared with those from the NF group, showed alterations in insulin-signaling pathway with reduced pIRS-1/IRS-1, pIRS-2/IRS-2, pAkt/Akt and PI3K protein expressions which could be associated with the insulin resistance and glucose intolerance observed in this group. Similar data have been observed in a rat postnatal OF model^25^ associated with OS and in Zucker fatty rats^64^.

An important limitation in our study is that the livers from OF and NF groups were collected in a fasting condition, while insulin-signaling changes are more accurately evaluated under stimulated conditions. Although our results are significant even in the absence of postprandial stimulation, they should be carefully interpreted. Glucose is the main stimulus for insulin secretion. Glucose enters cells by means of several glucose transporter proteins (GLUTs) in an ATP-independent manner. GLUT-4 plays a major role in glucose transport in adipose tissue and striated muscle and, consequently, in its metabolism. In the liver, however, GLUT-4 levels are considered very low and not relevant to hepatic insulin signaling. On the contrary, GLUT-2 is highly expressed in liver and does not undergo insulin-stimulated translocation^65^. We measured only the total hepatic levels of GLUT-4 and GLUT-2. At 7 months of life, in livers from the OF group compared with those from NF group, we observed an unexpected increase in total GLUT-4 protein and a decrease in GLUT-2 protein expression levels. While no conclusion can be drawn regarding the consequences of increased GLUT-4 signaling in the liver, a decrease in GLUT-2 protein expression may lead to alter insulin signaling in the liver. Recently, data have suggested that changes in GLUTs expression levels may be surrogate markers of hepatocyte senescence and may be associated with liver disease^66^. SIRT-1 plays a major role in the regulation of hepatic metabolism through its deacylase activity and *via* its direct and indirect involvement in insulin signaling^67^. SIRT-1 deficiency has been linked to hepatic glucose overproduction and chronic hyperglycemia^68^, as well as insulin resistance and metabolic syndrome^69^. Therefore, impaired SIRT-1 expression and activity could be associated with the metabolic disorders observed in the OF group at adulthood. No difference appeared between the groups concerning the expression of these two glucose transporters at PND 24.

In conclusion, this study demonstrates that transient postnatal OF leads to OS-induced hepatocyte SIPS associated with decreased SIRT-1 activity and expression at adulthood. We included in this study only males and therefore, it would be interesting to observe whether early transient postnatal OF during lactation period could have a gender effect on hepatic disorders described in this study. The reduced SIRT-1 functionality could be the consequence of OS and is likely linked to hepatic fibrosis, microsteatosis and impaired hepatic insulin signaling and glucose transporters, leading to insulin resistance and glucose intolerance observed at adulthood. Such hepatic dysfunctions could be a mark of later associated with the development of non-alcoholic fatty liver disease. Therefore, modulating SIRT-1 expression using resveratrol or caloric restriction may be a potential intervention in view of reversing hepatic dysfunctions at adulthood, secondary to altered metabolic programming induced by a transient early postnatal OF. The results of this study also demonstrate that a transient postnatal OF on breast milk is responsible for major changes in body composition and glucose/insulin metabolic characteristics. They suggest that early nutrition has a particular importance during the period developmental programming in early infancy.

## Methods

### Animal model

Investigations were performed in accordance with Directive 2010/63/EU of the European Parliament and the Guidelines for the Care and Use of Laboratory Animals published by the US National Institutes of Health (NIH Publication No. 85–23, revised 1996). The Comité d’Ethique de l’Experimentation Animale, Université de Bourgogne-Franche-Comté, Dijon, France, acted as the institutional review board (protocol agreement number: 00412.03) and specifically approved this study. Throughout the procedure, care was taken to avoid animal suffering and to ensure animal welfare, e.g., by improving the cage environment. Throughout the protocol, no animal died from the treatment or became moribund such that early sacrifice was necessary. We did not observe specific signs of pain or distress (e.g., abnormal animal behavior, decreased food or water consumption, prostration) throughout the study.

Adult female C57BL/6 mice (Charles River, L’Arbresle, France) from 6 weeks of age were individually housed. After one week of adaptation, they were mated overnight with males at a proportion of 2:1. The females were then housed in individual cages for gestation and lactation and had free access to tap water. They were kept on a standard pellet diet (A03, SAFE Diets Augy, France) under a 12-h light-dark cycle in a room maintained at a controlled temperature of 22 °C and constant humidity. On the third day of life, male pups were randomly distributed among mothers to achieve cross fostering. Litter sizes were adjusted to 9 male pups for NF or reduced to 3 male pups to induce postnatal OF in the lactation period, thereby forming the NF and OF groups. Each litter included pups from one to six different dams to increase genetic variability within the litters. Excess pups were rapidly sacrificed by decapitation after a brief period of isoflurane anesthesia. After weaning, on PND 24, the mice in both groups had free access to a standard diet (A04, SAFE Diets Augy, France) and water. Throughout the life of the mice, body weight and food intake were measured weekly and then monthly.

Males (NF group, n = 8; OF group, n = 8) were specifically studied at PND 24 and at 7 months of age. For histological studies and evaluation of OS and accelerated senescence, mice were anesthetized after 6 h of fasting by an intraperitoneal injection of sodium pentobarbital (80 mg/kg). The liver was then harvested and immediately frozen/fixed in liquid nitrogen/formol until further processing.

### Glucose and insulin tolerance tests (GTTs and ITTs)

Intraperitoneal GTTs and ITTs were performed after a 5-h fasting period in a separate group of awake mice aged 6 months. GTTs consisted in an intraperitoneal injection of glucose (2 g/kg). Blood droplets were collected from the tail vein just prior to the glucose injection (time 0) and at 15, 30, 45, 60, 75, 90, 105 and 120 min following the injection. The blood glucose concentrations were measured using a glucometer and FreeStyle Optium test strips (Abbott, Santa Clara, CA, USA). For the ITTs, mice were injected with 0.75 IU/kg human recombinant insulin (Actrapid®, Novo Nordisk, France), and blood glucose concentration was determined at 0, 15, 30, 45, 60, 75, 90, 105 and 120 min thereafter. The data of GTT and ITT are represented as blood glucose concentration in mg/dL.

### Western blotting

Liver proteins from NF (n = 8) and OF (n = 8) groups were extracted at PND 24 and at 7 months of life (from the medial lobe of the snap-frozen livers) using 500 μl of RIPA buffer (pH 7.4, Tris–HCl 50 mM, NP-40 1%, sodium deoxycholate 0.25%, NaCl 150 mM, EDTA 1 mM, NaF 50 mM, Na_3_VO_4_ 1 mM and beta-glycerophosphate 25 mM) (Sigma-Aldrich, St. Louis, MO, USA). Prior to tissue homogenization, a mini protease inhibitor tablet (Roche Diagnostics, Indianapolis, IN, USA) was added to the lysis buffer. Homogenized tissue was left on ice for 5 min and then sonicated. Following sonication, the homogenate was centrifuged for 25 min at 14,000 rpm and 4 °C. The supernatant was retained for protein quantification (Pierce BCA Protein Assay Kit, Thermo Scientific, Rockford, IL, USA) and western blot analysis.

Denatured (10 min at 70 °C) liver proteins (20 μg) from the NF and OF groups were separated on the same gradient gel (NuPAGE 4–12% Bis-Tris gel, Thermo Scientific) and transferred for 2 h at room temperature to Whatman nitrocellulose membranes (Thermo Scientific). Ponceau staining (Thermo Scientific) confirmed the presence of proteins on the membranes. All primary antibody incubations were performed in blocking buffer (TBS-Tween 2%-bovine serum albumin (BSA) 3%) overnight at 4 °C. Antibodies against the insulin receptor substrates 1 and 2 (IRS-1, IRS-2), phospho-IRS-1 (pIRS-1), PI3 kinase (PI3K), phospho-Akt (Ser473) (pAkt), Akt, SOD Cu/Zn, catalase (CAT), glucose transporter-4 (Glut-4), glucose transporter-2 (Glut-2; Abcam Ltd., Cambridge, UK), SIRT-1, retinoblastoma tumor suppressor protein (Rb) and phospho-Rb (Ser807/811) (pRb), p21^WAF^, p53 and acetyl-p53 (Lys382), CDKN2A/p16^INK4a^ and pIRS-2 (Abcam), glucose-6-phosphate dehydrogenase (G6PDH; Cell Signaling Technology (Danvers, MA, USA)) and alpha-smooth muscle actin (α-SMA; Sigma-Aldrich) were purchased and used at the dilutions recommended for immunoblotting (1:1000). Incubations with anti-mouse or anti-rabbit secondary antibodies (1/2000; Cell Signaling) were performed for 1 h at room temperature in blocking buffer (TBS-Tween 2%-BSA 3%). The antibodies were visualized using enhance chemiluminescence western blotting substrate (Thermo Scientific). A G-BOX Imaging System (GeneSys, Syngene, Cambridge, UK) was used to detect specific bands, and the optical density of each band was measured using specific software (GeneTools, Syngene) for all blots. However, for pRb/Rb, pAkt/Akt and SOD Cu/Zn, pIRS-2/IRS-2 proteins detection at 7 months of life, the revelation has been realized using photographic film (CL-XPosure ^TM^ Film, Thermo Scientific), scanned and the optical density of each band was measured using specific software (GeneTools). Representative experiments are shown for each protein.Full-length western blots are represented in supplementary information (supplemental data 1 and 2).

### Superoxide anion (O_2_^•−^) detection using chemiluminescence

Liver O_2_^•−^ production was evaluated in NF (n = 8) and OF (n = 8) groups at PND 24 and at 7 months of life using the oxidative fluorescent dye hydroethidine (2 μM, Sigma-Aldrich)^70^ in comparisons to negative control (autofluorescence detection). In the presence of O_2_^•−^, hydroethidine is oxidized to fluorescent ethidium bromide, which is trapped in DNA by intercalation. Deparaffinized hepatic sections (5-μm thick) were stained with hydroethidine by incubating in a light-protected humidified chamber at 37 °C for 30 min. The sections were rinsed with phosphate-buffered saline (PBS; 2 times for 10 min) and mounted using Fluoromount-G mounting medium with 4′6-diamidino-2-phenylindole (DAPI; Interchim, France). Images were obtained using a laser scanning confocal microscope (Leica SP5) equipped with an argon laser. Fluorescence was detected from at least 4 hepatic sections per animal with a 514-nm long-pass filter and evaluated with ImageJ software (http://rsbweb.nih.gov/ij), and autofluorescence of liver was subtracted.

### Oxidative DNA damage detection

Deparaffinized liver sections (5-μm thick) from 7-month-old mice in the NF (n = 8) and OF (n = 8) groups were stained with 53BP-1 (1/100, Abcam) overnight at 4 °C. The sections were then washed with 1x PBS (2 times for 10 min) and incubated for 1 h with Alexa Fluor-488-conjugated donkey anti-rabbit IgG (1/200, Abcam). The sections were then rinsed with 1x PBS (2 times for 10 min) and mounted using Fluoromount-G mounting medium with DAPI (Interchim). A negative control was obtained using incubation only with secondary antibody. Fluorescence from at least 4 hepatic sections per animal was evaluated using ImageJ software.

### Histological analysis

At PND 24 and 7 months, livers from NF (n = 8) and OF (n = 8) groups were rapidly removed and fixed for 24–48 h in 4% formol. Equatorial cross-sections were paraffin-embedded, and 5-μm sections were stained with H&E for the hepatic structure evaluation. In addition, livers were stained with Masson’s Trichrome to evaluate hepatic fibrosis. For all histological analyses, three images were captured for each animal. A pathologist (Prof. C. Sempoux) confirmed the histological observations. Hepatic fibrosis was quantified using ImageJ software. A quantitative analysis was performed by a single examiner (C.Y). For each image in OF and NF groups, a “stack image” was generated, and a color threshold was applied to identify the stained structure. The results are reported as the percentage of fibrotic area of total area.

### Histological detection of cell senescence

The presence of cytoplasmic accumulation of highly oxidized insoluble proteins, known as lipofuscin staining has been considered a marker of SIPS^35^. Lipofuscin staining was identified using the following histological analyses: d-PAS, Fontana-Masson and SBB staining. For all histological analyses, three images were captured for each animal. Lipofuscin staining was quantified using ImageJ software. A quantitative analysis was performed by a single examiner (C.Y). For Fontana-Masson and SBB staining, each image in OF and NF groups, a “stack image” was generated, and a color threshold was applied to identify the stained structure. The results are reported as the percentage of lipofuscin area of total area.

### Statistical analyses

All data were presented as mean ± SD. Experimental observations were analyzed using Mann-Whitney U test (western blot) and student t test. GraphPad Prism 7 (La Jolla, CA, USA) was used for performing statistical analyses and creating graphics. The significance level was set at p < 0.05.

### Data availability

All data generated or analysed during this study are included in this published article.

## Electronic supplementary material

Full-length blots
