# Supplementary material for: Transient postnatal overfeeding causes liver stress-induced premature senescence in adult mice
Source: Sci Rep. 2017 Oct 10;7:12911. doi: 10.1038/s41598-017-11756-2 (PMC5635041; doi:10.1038/s41598-017-11756-2)
Supplement: Supplementary file 1 — Full-length blots [file 41598_2017_11756_MOESM1_ESM.pdf]

Transient postnatal overfeeding causes liver stress-induced premature senescence in adult mice

Catherine Yzydorczyk, Na Li, Hassib Chehade, Dolores Mosig, Mickael Bidho, Basile Keshavjee, Jean Baptiste Armengaud, Katya Nardou, Benazir Siddeek, Mohamed Benahmed, Catherine Vergely, Umberto Simeoni

Supplemental data 1

The whole full-length western blots at 7 months of age concerning Catalase (A), SOD Cu/Zn (B), G6PDH (C), p21 (D), SIRT-1 (E), Acp53/p53 (F), p16 (G), pRb/Rb (H), alpha-SMA (I), pIRS-1/IRS-1 (J), pIRS-2/IRS-2 (K), PI3K (L), pAKT/AKT (M), GLUT-4 (N), GLUT-2 (O) protein levels. Square red correspond to selected bands in the manuscript.

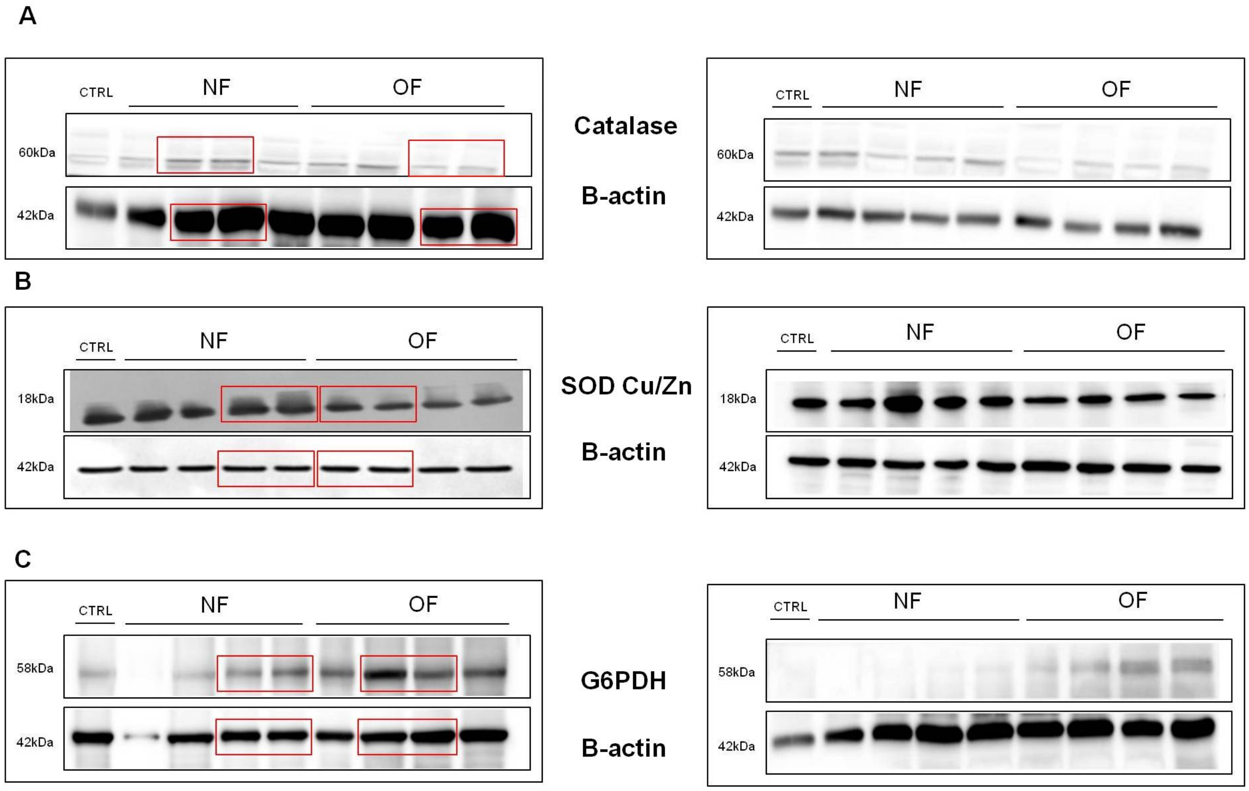
A
CTRL
NF
OF
60kDa
42kDa
Catalase
B-actin
B
18kDa
42kDa
SOD Cu/Zn
B-actin
C
58kDa
42kDa
G6PDH
B-actin

D

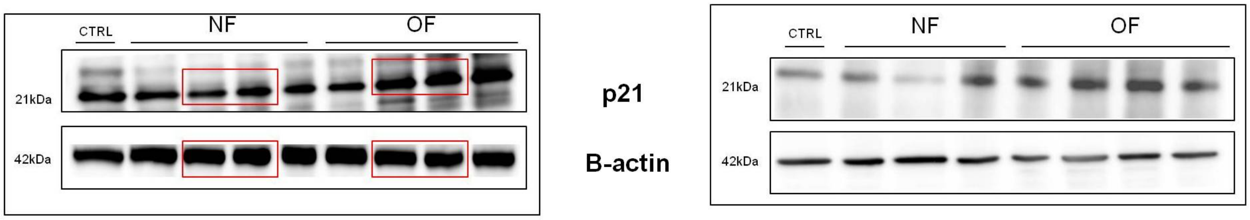
CTRL
NF
OF
21kDa
42kDa
p21
B-actin

E

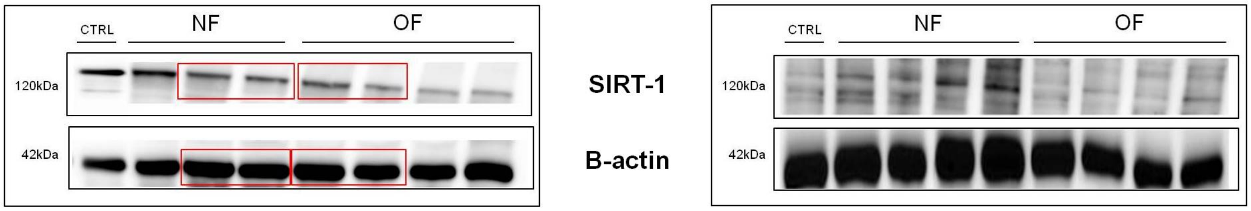
CTRL
NF
OF
120kDa
42kDa
SIRT-1
B-actin

F

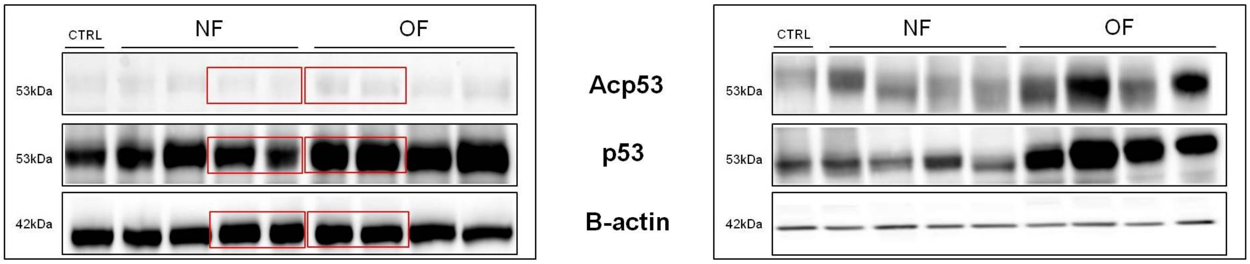
CTRL
NF
OF
53kDa
42kDa
Acp53
p53
B-actin

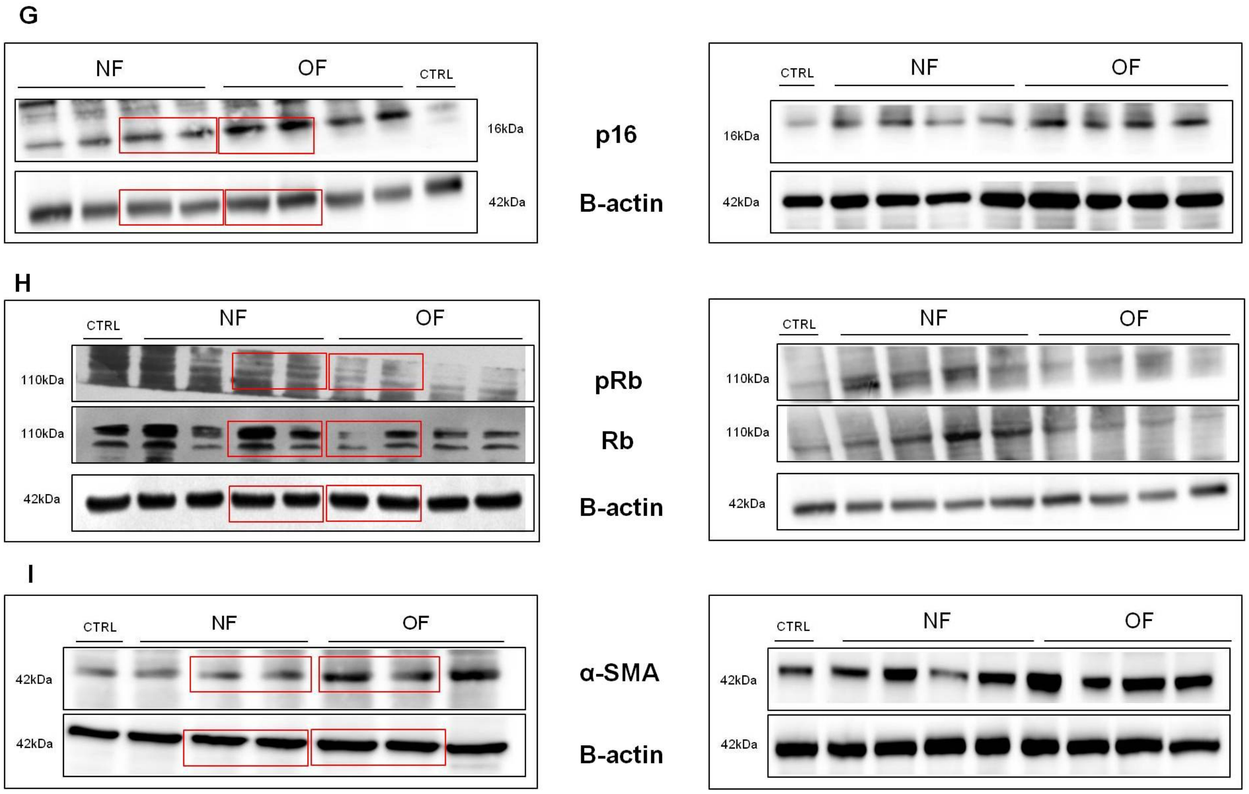
G
NF
OF
CTRL
16kDa
42kDa
p16
B-actin
H
CTRL
NF
OF
110kDa
110kDa
42kDa
pRb
Rb
B-actin
I
CTRL
NF
OF
42kDa
42kDa
α-SMA
B-actin

J

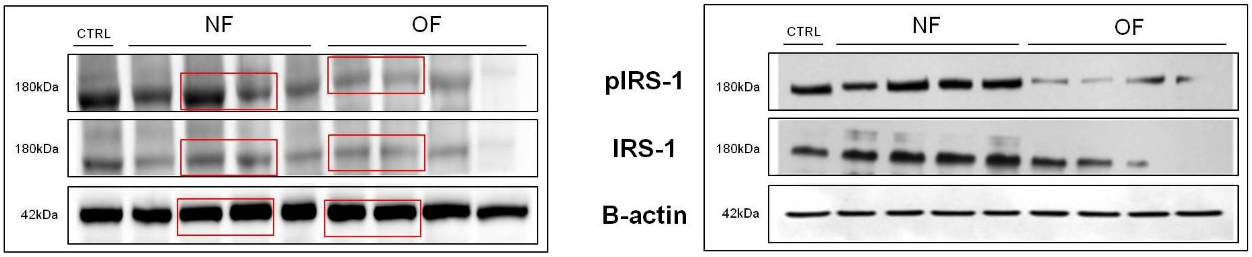
CTRL
NF
OF
180kDa
180kDa
42kDa
pIRS-1
IRS-1
B-actin
CTRL
NF
OF
180kDa
180kDa
42kDa

K

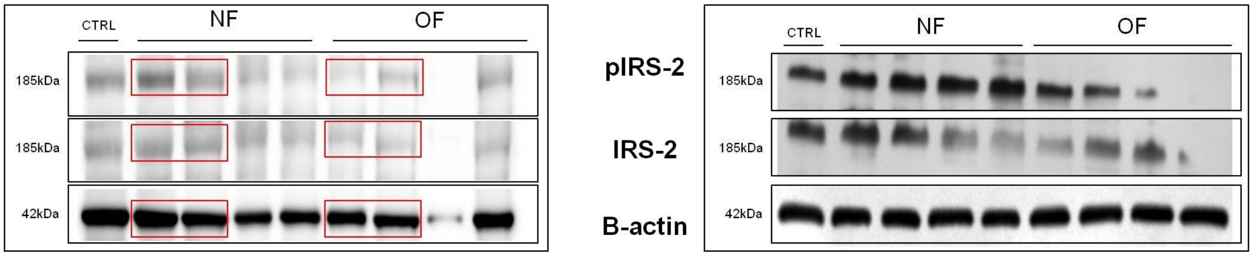
CTRL
NF
OF
185kDa
185kDa
42kDa
pIRS-2
IRS-2
B-actin
CTRL
NF
OF
185kDa
185kDa
42kDa

L

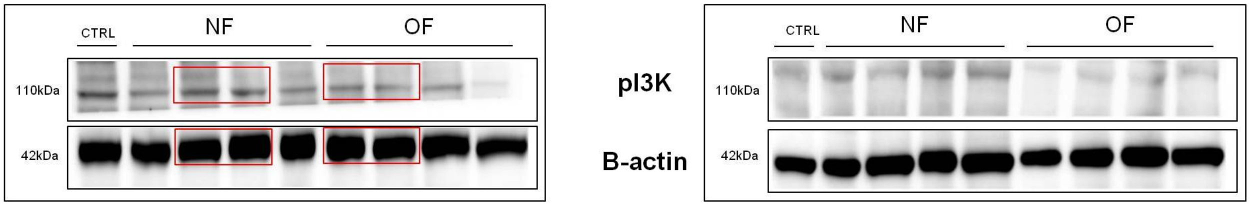
CTRL
NF
OF
110kDa
42kDa
pI3K
B-actin
CTRL
NF
OF
110kDa
42kDa

M

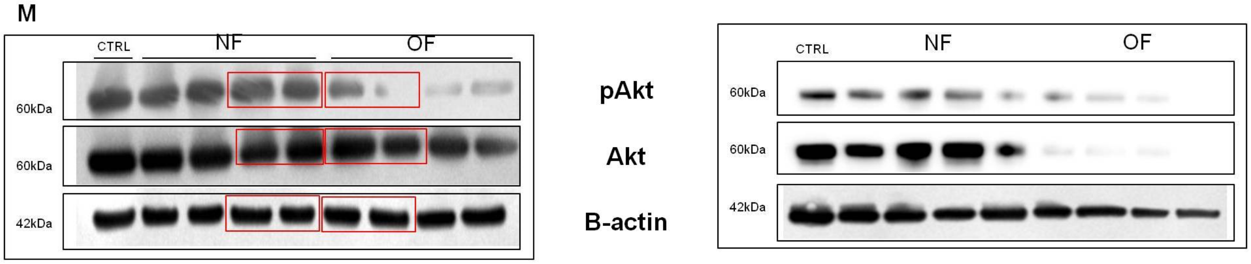
CTRL
NF
OF
60kDa
60kDa
42kDa
pAkt
Akt
B-actin
CTRL
NF
OF
60kDa
60kDa
42kDa

N

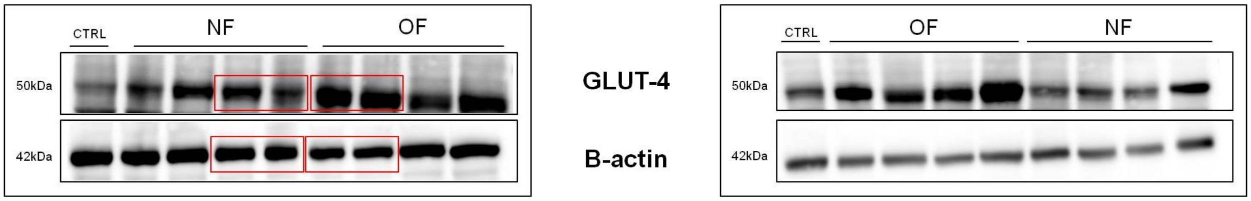
CTRL
NF
OF
50kDa
42kDa
GLUT-4
B-actin
CTRL
OF
NF
50kDa
42kDa

O

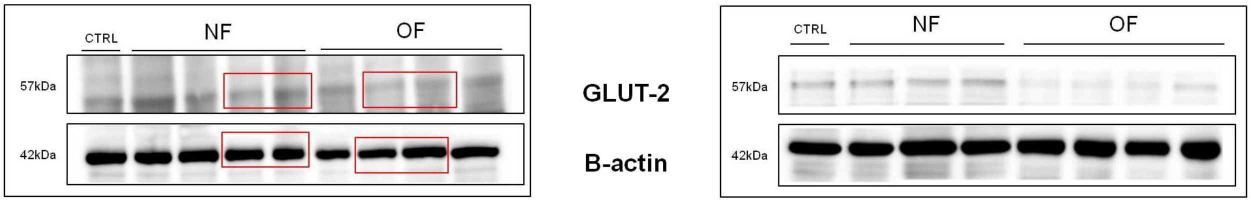
CTRL
NF
OF
57kDa
42kDa
GLUT-2
B-actin
CTRL
NF
OF
57kDa
42kDa

Supplemental data 2

The whole full-length western blots at PND24 concerning Catalase (A), SOD Cu/Zn (B), G6PDH (C), p21 (D), SIRT-1 (E), Acp53/p53 (F), p16 (G), pRb/Rb (H), pIRS-1/IRS-1 (I), pIRS-2/IRS-2 (J), PI3K (K), pAKT/AKT (L), GLUT-4 (M), GLUT-2 (N) protein levels. Square red correspond to selected bands in the manuscript.

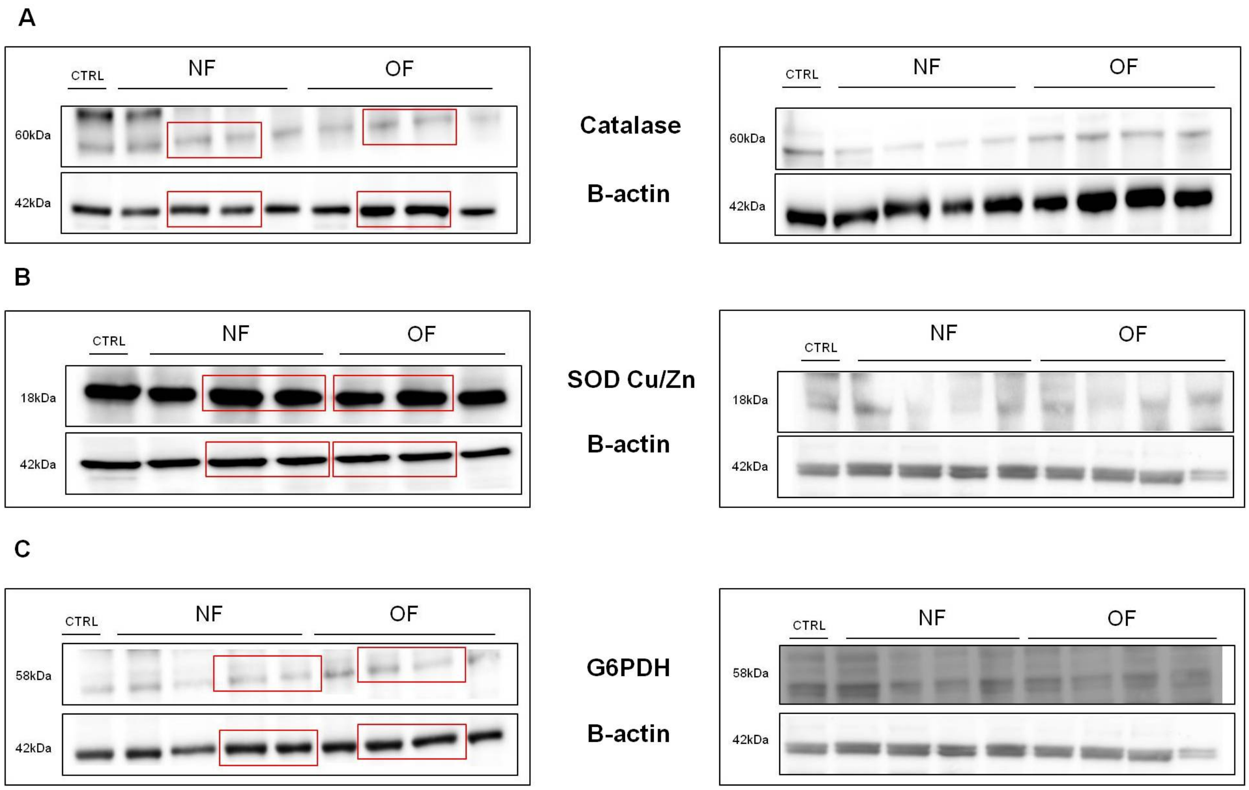

A
CTRL
NF
OF
60kDa
42kDa
Catalase
B-actin
B
18kDa
42kDa
SOD Cu/Zn
B-actin
C
58kDa
42kDa
G6PDH
B-actin

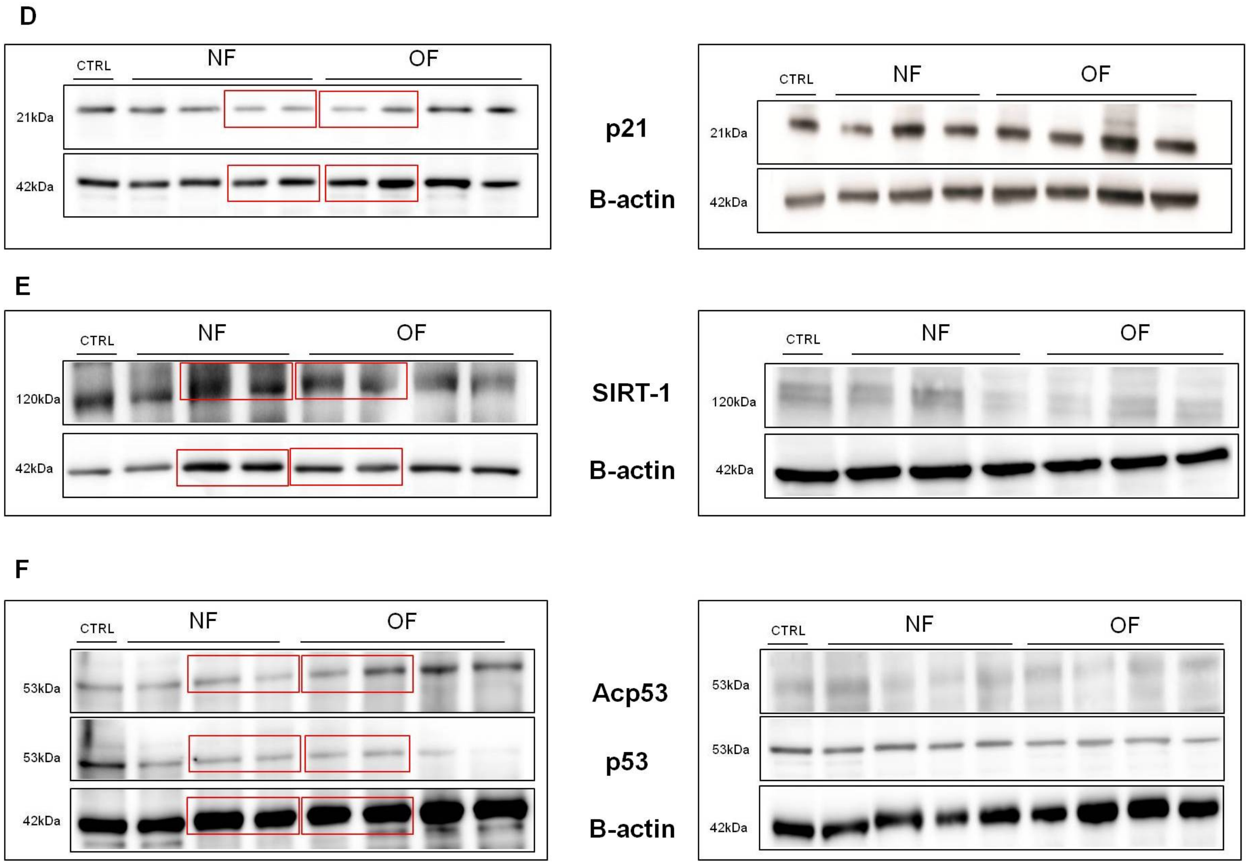
D
CTRL
NF
OF
21kDa
42kDa
p21
B-actin
E
CTRL
NF
OF
120kDa
42kDa
SIRT-1
B-actin
F
CTRL
NF
OF
53kDa
53kDa
42kDa
Acp53
p53
B-actin

G

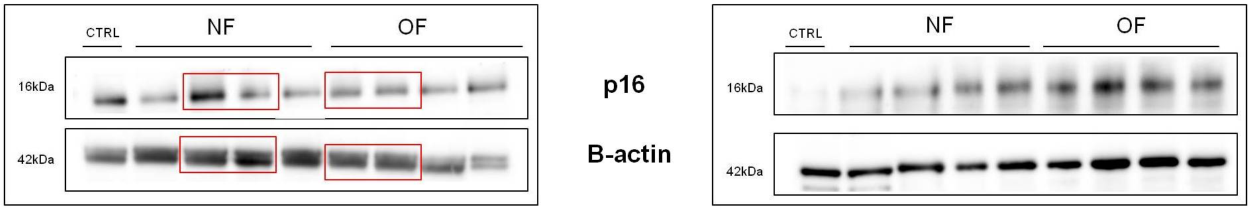
CTRL
NF
OF
16kDa
42kDa
p16
B-actin
CTRL
NF
OF
16kDa
42kDa

H

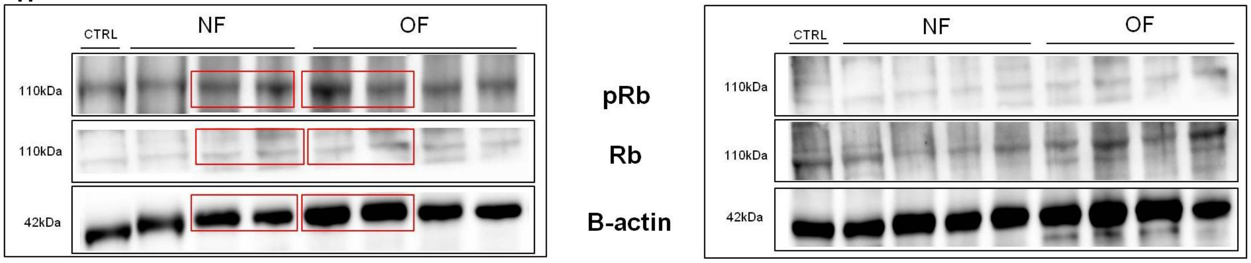
CTRL
NF
OF
110kDa
110kDa
42kDa
pRb
Rb
B-actin
CTRL
NF
OF
110kDa
110kDa
42kDa

I

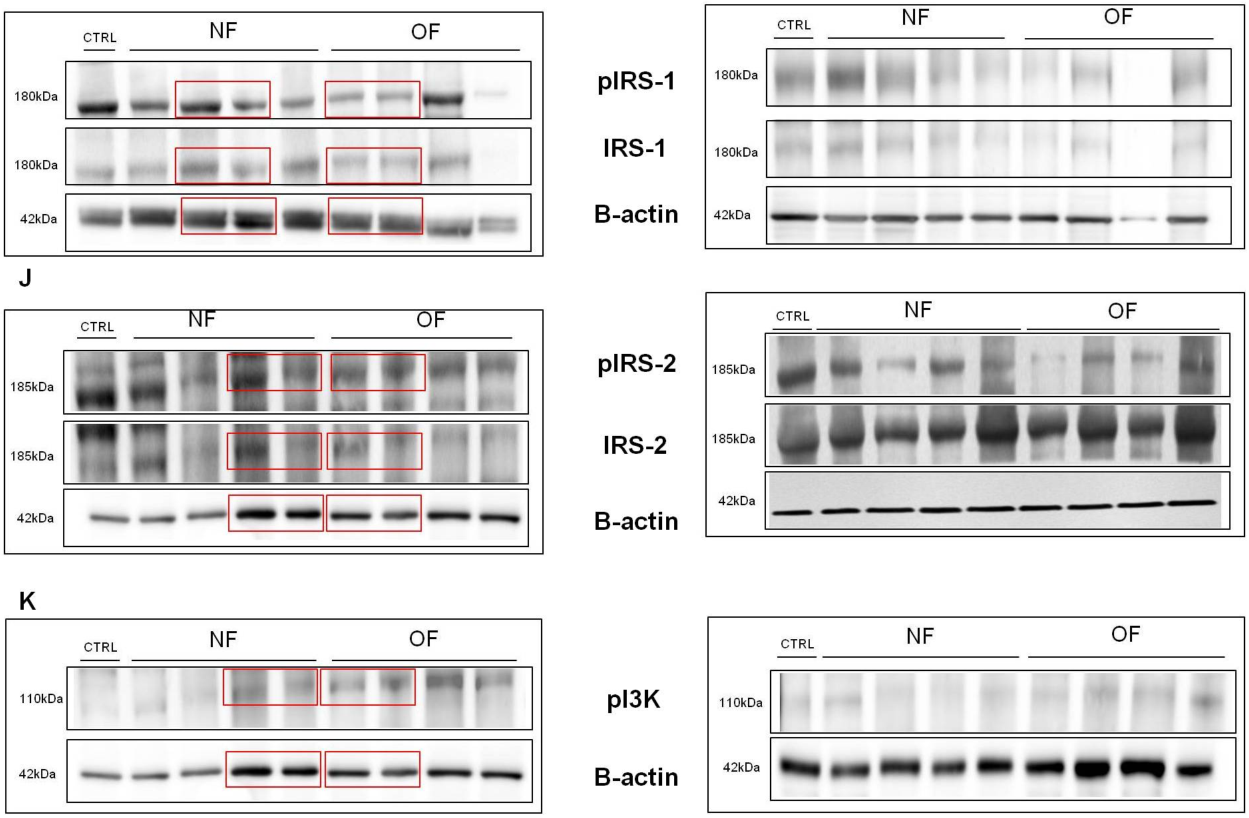
CTRL
NF
OF
180kDa
180kDa
42kDa
pIRS-1
IRS-1
B-actin
J
185kDa
185kDa
42kDa
pIRS-2
IRS-2
B-actin
K
110kDa
42kDa
pI3K
B-actin

L

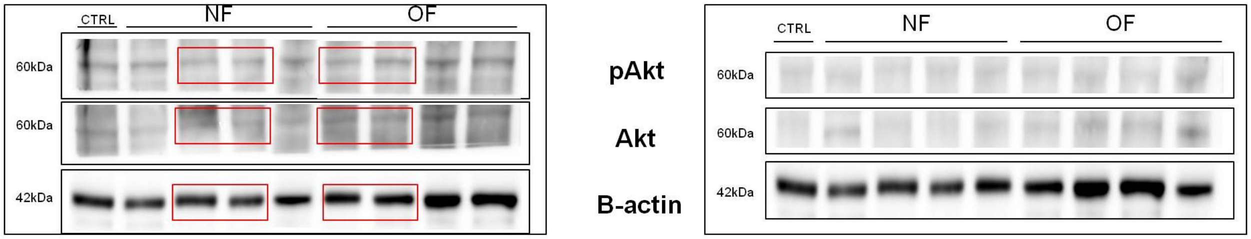
CTRL
NF
OF
60kDa
60kDa
42kDa
pAkt
Akt
B-actin
CTRL
NF
OF
60kDa
60kDa
42kDa

M

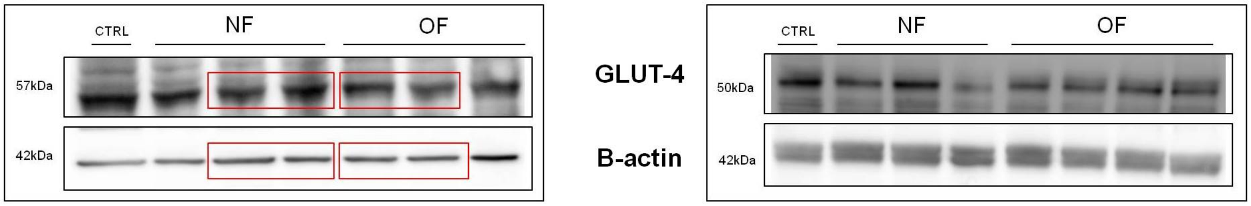
CTRL
NF
OF
57kDa
42kDa
GLUT-4
B-actin
CTRL
NF
OF
50kDa
42kDa

N

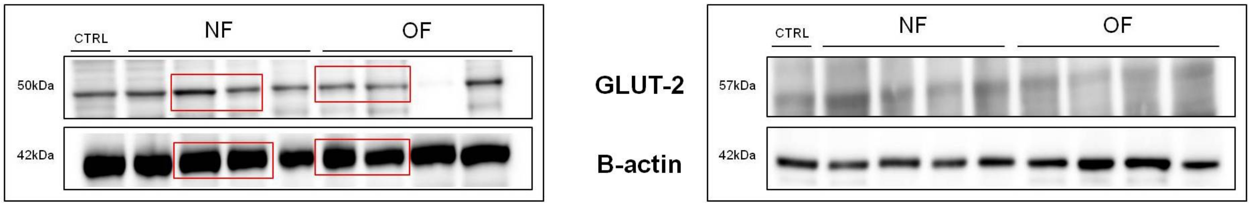
CTRL
NF
OF
50kDa
42kDa
GLUT-2
B-actin
CTRL
NF
OF
57kDa
42kDa
